# UVB Induces a Genome-Wide Acting Negative Regulatory Mechanism That Operates at the Level of Transcription Initiation in Human Cells

**DOI:** 10.1371/journal.pgen.1004483

**Published:** 2014-07-24

**Authors:** Ákos Gyenis, David Umlauf, Zsuzsanna Újfaludi, Imre Boros, Tao Ye, Làszlò Tora

**Affiliations:** 1 Cellular signaling and nuclear dynamics program, Institut de Génétique et de Biologie Moléculaire et Cellulaire, Illkirch, France; 2 Centre National de la Recherche Scientifique, UMR7104, Illkirch, France; 3 Institut National de la Santé et de la Recherche Médicale, U964, Illkirch, France; 4 Université de Strasbourg, Illkirch, France; 5 University of Szeged, Faculty of Sciences and Informatics, Department of Biochemistry and Molecular Biology, Szeged, Hungary; 6 Microarrays and deep sequencing platform, Institut de Génétique et de Biologie Moléculaire et Cellulaire, Illkirch, France; University of Texas at Austin, United States of America

## Abstract

Faithful transcription of DNA is constantly threatened by different endogenous and environmental genotoxic effects. Transcription coupled repair (TCR) has been described to stop transcription and quickly remove DNA lesions from the transcribed strand of active genes, permitting rapid resumption of blocked transcription. This repair mechanism has been well characterized in the past using individual target genes. Moreover, numerous efforts investigated the fate of blocked RNA polymerase II (Pol II) during DNA repair mechanisms and suggested that stopped Pol II complexes can either backtrack, be removed and degraded or bypass the lesions to allow TCR. We investigated the effect of a non-lethal dose of UVB on global DNA-bound Pol II distribution in human cells. We found that the used UVB dose did not induce Pol II degradation however surprisingly at about 93% of the promoters of all expressed genes Pol II occupancy was seriously reduced 2–4 hours following UVB irradiation. The presence of Pol II at these cleared promoters was restored 5–6 hours after irradiation, indicating that the negative regulation is very dynamic. We also identified a small set of genes (including several p53 regulated genes), where the UVB-induced Pol II clearing did not operate. Interestingly, at promoters, where Pol II promoter clearance occurs, TFIIH, but not TBP, follows the behavior of Pol II, suggesting that at these genes upon UVB treatment TFIIH is sequestered for DNA repair by the TCR machinery. In agreement, in cells where the TCR factor, the Cockayne Syndrome B protein, was depleted UVB did not induce Pol II and TFIIH clearance at promoters. Thus, our study reveals a UVB induced negative regulatory mechanism that targets Pol II transcription initiation on the large majority of transcribed gene promoters, and a small subset of genes, where Pol II escapes this negative regulation.

## Author Summary

Our genome is continuously exposed to genotoxic attacks that generate aberrant DNA structures. These can block the transcribing DNA-dependent RNA polymerase II (Pol II) enzyme and can lead to deleterious cellular processes. Cells have developed several mechanisms to stop Pol II, repair the roadblocks and to restore normal polymerase traffic. Numerous efforts investigated the fate of blocked Pol II during DNA repair mechanisms and suggested that stopped Pol II complexes can either backtrack, be removed or bypass the lesions to allow repair. We carried out a genome-wide analysis of Pol II behavior upon a DNA damaging stress, UVB, which is relevant from the public health standpoint. Thus, we could follow UVB-induced Pol II behavior changes on every human gene over time. We uncovered a novel UV induced negative regulatory mechanism, which inhibits the recruitment of Pol II to the promoters of about 93% of all transcribed genes, and a small subset of gene (including regulators of repair, cell growth and survival) that escapes this negative regulation, probably because their gene products are required during/after UVB irradiation. Thus, we uncover how a cell induces a global negative regulation at the level of transcription initiation in response to a genotoxic stress.

## Introduction

Proper cell homeostasis and function requires expression of the DNA encoded information. Maintenance of genome integrity and accurate replication is crucial for correctly regulated gene expression. Transcription of thousands of coding and non-coding RNAs by the RNA polymerase II (Pol II) is a regulated multistep process that can be divided into five stages: pre-initiation, initiation, promoter clearance, elongation and termination. Based on numerous genome-wide studies analyzing Pol II transcription in several metazoan organisms using chromatin immunoprecipitation followed by deep sequencing (ChIP-seq) it is now clear that on different regions of an expressed gene, distinct types of Pol II occupancy signals can be detected. The “canonical” Pol II occupancy ChIP-seq profile on an average expressed gene displays Pol II molecules engaged in the major phases of transcription [1], [2], [3], [4], [5], [6], [7] and can be divided in three major regions: i) the sharp and usually high peak centered about +50 bp downstream of the transcription start site (TSS), representing Pol II molecules that have entered the pre-initiation complex (PIC) during transcription initiation/clearance and stopped at promoter proximal pausing position. Analyses of short transcribed RNA molecules showed that these arrested polymerases are predominantly in a transcriptionally engaged state [4], [8], [9]; ii) the background-like low signals in the gene body (GB), representing quickly elongating Pol II molecules; and iii) the broad signal downstream from the 3′ end of the annotated genes (EAGs) representing Pol IIs that have finished transcribing the pre-mRNA and are slowly transcribing and approaching the termination site often 4–6 kb away from the 3′end of the gene [10], [11], [12], [13] (see also below).

Damage or alterations of the DNA structure can threaten the progression of transcription. Indeed, Pol II driven transcription has been reported to be disturbed by “roadblocks” on the DNA template, which arises from both environmental and endogenous sources, such as special DNA sequences, non-canonical DNA structures, topological constrains and DNA lesions [14].

UV light is one of the most genotoxic environmental sources of transcription-blocking DNA damages. Different wavelengths of the UV light can generate a wide range of lesions in the DNA template. Based on its wavelength, UV light can be divided into UVA (315–400 nm), UVB (280–315 nm) and UVC (<280 nm). UVC has higher energy level than UVA and UVB due to its shorter wavelength; however, UVC radiation is not relevant from a public health standpoint as it is absorbed by the ozone layer. UV-related lesions in living organisms are induced mostly by UVA and UVB.

The ionizing energy of UVB and UVC radiation is directly absorbed by the DNA and can cause cyclobutane pyrimidine dimers (CPDs), pyrimidine 6-4 pyrimidone photoproducts (6-4PPs), which are the most persistent and predominant lesions [15]. Nucleotide excision repair (NER) is a mechanism that removes UV-induced lesions efficiently using two distinct pathways [16], [17]: The first, called transcription coupled repair (TCR), is mainly linked to Pol II transcription and is highly specific and efficient. TCR preferentially removes DNA lesions from the transcribed strand of active genes, allowing blocked transcription to resume. In transcribed regions UV-induced DNA lesions are known to block the elongating Pol II complex causing persistent transcriptional arrest. CPDs arrest transcription as they occupy the active site of Pol II. Moreover, DNA crosslinked proteins and interstrand crosslinks block transcription by steric hindrance before the lesion reaches the active site of Pol II. When the elongating polymerases arrest at a damage site, TCR is triggered and the corresponding lesions are repaired. One of the first steps of the TCR consist of the recognition of the blocked Pol II elongation complex by CSB (Cockayne syndrome b protein, also called ERCC6), which will trigger the further recruitment of NER factors essential to carry out the repair process. Amongst those factors is the general transcription factor TFIIH that plays a role both in repair and in Pol II transcription initiation [18], [19], [20]. Global genome repair (GGR) is the second pathway of NER that mainly acts on intergenic or non-coding regions of the genome and recognizes DNA lesions based on their base-pairing and helix-disrupting properties. GGR for some types of lesions (such as UV-induced CPDs) is less efficient than the repair carried out by the TCR machinery on the transcribed strand of active genes [21], [22].

*In vitro* the half-life of an arrested Pol II at a CPD can be ∼20 hours, and it covers 10 nt upstream and 25 nt downstream of the CPD [23], [24]. Therefore the arrested Pol II seems to create a roadblock for all the transcription on the given open reading frame, and may also block the access of the lesion by repair factors [25], [26]. Such persistent Pol II blocks and subsequent transcriptional arrest can initiate checkpoint signaling, which can lead to cellular apoptosis [27]. Moreover, the stalled Pol II at a helix-distorting DNA damage can block the access of the nucleotide excision repair factors to the lesions [28].

It seems that cells have evolved several solutions to deal with the persistently stopped Pol II elongation complexes: i) Pol II can bypass the lesions, but this process is slow and extremely inefficient [29], ii) CSB removes Pol II from the lesions through its Swi/Snf-like activity [19], iii) Pol II is backtracking allowing repair [30]. The restart of transcription depends on the cleavage and reposition the 3′ end of the RNA to the active center of Pol II, which can be mediated by the TFIIS elongation factor [31]. Persistent DNA damage can also result in the poly-ubiquitination and degradation of the largest subunit of Pol II [26]. This would make the lesion accessible for other repair processes and allow a new round of Pol II transcription. It has been suggested that the Pol II degradation pathway is activated only when the transcription activity of the blocked Pol II cannot be restored, and this pathway is an alternative to TC-NER.

Our knowledge about Pol II transcription inhibition after UV irradiation is based on studies carried out on a few model genes and in experiments often using lethal UVC doses. Moreover, many studies have investigated the fate of Pol II elongation complexes during TCR, but very little information has been obtained on how TCR influences the whole Pol II transcription cycle during TCR. Therefore, the mechanism by which Pol II transcription is affected genome-wide upon sublethal doses of UV irradiation is not yet well understood. To investigate the fate of Pol II during TCR processes, we have investigated Pol II occupancy at a genome wide level following UVB treatment over time in human MCF7 cells. Our results show that on about 93% of the promoters of expressed genes Pol II occupancy is seriously reduced 2–4 hours following UVB irradiation, and that the presence of Pol II is restored to “normal”, or even sometimes higher, levels 5–6 hours after irradiation. We also identified a smaller set of genes, where the presence of Pol II at the promoter regions does not decrease, but rather increases at the promoters and also throughout the entire transcription units of these genes after UVB irradiation. Thus, our study reveals a global negative regulatory mechanism that targets RNA polymerase II transcription initiation on the large majority of transcribed genes following sublethal UV irradiation and a small subset of key regulatory genes, where Pol II escapes the negative regulation.

## Results

### Effect of a sublethal dose of UVB irradiation on RNA synthesis in MCF7 cells

To investigate the general effect of UVB irradiation on transcription in human cells, we have set up to find irradiation conditions that do not induce apoptosis and under which cells can repair UV-lesions. To define a sublethal irradiation dose, we carried out a survival assay during which we tested the effect of 55, 100 and 200 J/m^2^ of UVB irradiation on the DNA damage response proficient MCF7 human breast cancer cell line, containing wild type p53 (Figure S1, and see below). Upon 55, 100 and 200 J/m^2^ irradiation 95%, 50% and 30% of the plated cells survived the treatment, respectively (Figure S1A). Thus, for our further study we have chosen the 55 J/m^2^ dose of UVB. Importantly, 55 J/m^2^ UVB induces the main UV-DNA lesions as we detected the presence of CPDs with an anti-CPD antibody up to 24 hours after 55 J/m^2^ UVB irradiation (Figure S1B). Moreover, by testing the induction of DNA-damage response markers, such as phospho-Chk1 and phospho-p53, our experiments show that the 55 J/m^2^ UVB dose is enough to trigger the UV/DNA damage response of MCF7 cells (Figure S1C). Thus, throughout the study we used this sublethal UVB dose to study transcription in MCF7 cells.

**Figure 1:**
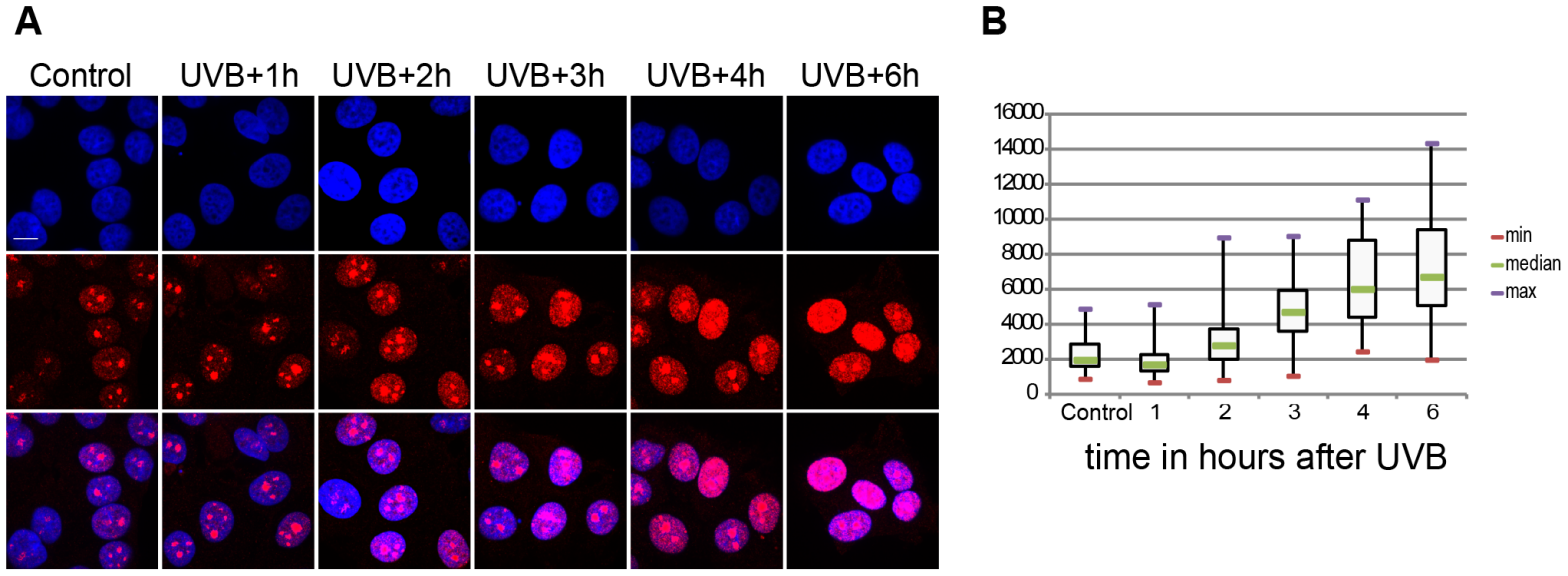
Global ongoing transcription in MCF7 cells upon UVB irradiation. (A) Incorporation of 5-fluorouridine (5-FU) in nascent transcripts was assessed in untreated or UV irradiated cells by indirect immunofluorescence using an anti 5-FU antibody. Cell nuclei were counterstained using DAPI and the overlay between the DAPI and 5-FU signal is shown. (B) 5-FU signal intensity in the nucleus (excluding the nucleolar signal) was measured in n randomly chosen cell nuclei using the ImageJ software (NIH), where n is 59 in control, 54 in UVB+1 h, 84 in UVB+2 h, 69 in UVB+3 h, 58 in UVB+4 h, 57 in UVB+6 h. Results are presented as a boxplot where the min, max and median values are in red, purple and green respectively. P values (non-equal variance), calculated by comparing the non-irradiates sample (control) with the other time points following UVB irradiation, are the following: UVB+1 h 0,00126; UVB+2 h 0,03203; UVB+3 h 1,91441 E-13; UVB+4 h 5,72028 E-17; UVB+6 h 9,452619 E-20.

It has been shown that UVC irradiation temporarily arrests Pol II transcription in human cells ([32], [33] and references therein). We investigated whether the above-defined 55 J/m2 UVB dose has the same effect on the global transcription program of MCF7 cells. To this end we assessed 5 fluorouridine (5FU) incorporation in the cells by immunofluorescence as a marker of newly synthesized RNA and ongoing transcription by all three RNA polymerases. To this end 5FU was added to each sample 20 min before harvesting the cells (Figure 1). This assay revealed a reduction in the levels of nascent transcripts 1 hour following irradiation and from three hours to six hours a constant increase in the global transcription levels (Fig. 1A and B). Surprisingly, at six hours following UVB irradiation nascent transcript levels increased about three times over the non-irradiated levels (Fig. 1A and B). Such unexpectedly strong nascent RNA production overshoots have already been described in MCF7 cells after estradiol stimulation and in other systems, and when combined with time dependent RNA degradation analyses, was suggested to shape transient physiological responses with precise mRNA timing and amplitude [34], [35]. Note however, that the used method labels all transcripts at a single cell level produced by the three RNA polymerases, including many non-coding transcripts, abortive transcripts produced during promoter clearance, and also short upstream antisense transcription start site associated RNAs (TSSa-RNAs) and others [36]. Nevertheless, our UVB irradiation experiment indicates that global nascent transcription is first inhibited by the used sublethal dose and then restarts again, suggesting that arrested polymerases may resume global transcription quickly as transcription-coupled repair is completed on the genome.

### Genome-wide Pol II occupancy is reorganized upon the sublethal dose of UVB irradiation

As TCR has been mainly linked to Pol II transcription [18] we investigated the effect of UVB irradiation on genome-wide Pol II behavior. The great advantage of mapping Pol II occupancy across the genome is that it may directly reflect transcriptional activity, unlike the measurement of mRNA levels at steady state, which are the cumulative result of numerous co-transcriptional and post-transcriptional processes. To map Pol II occupancy changes genome-wide following UVB irradiation, we carried out chromatin immunoprecipitation (ChIP) coupled to high throughput sequencing (seq) analyses using an antibody that recognizes the N-terminus of the largest subunit of Pol II (Rpb1) (N-20). MCF7 cells were either not irradiated, or treated with 55 J/m^2^ UVB and harvested 1, 2, 3, 4, 5 and 6 hours following irradiation. Cells were crosslinked with formaldehyde, ChIP was carried out and the recovered DNA fragments were deep sequenced. Specific Pol II bound sequence-reads were mapped to the human genome, and unique reads were considered for further analyses. For the comparative ChIP-seq analyses, all the seven datasets were normalized based on background tag densities calculated on intergenic regions (see Materials and Methods). Note that our control non-irradiated data set was very comparable to that obtained in MCF7 cells by [13].

**Figure 2:**
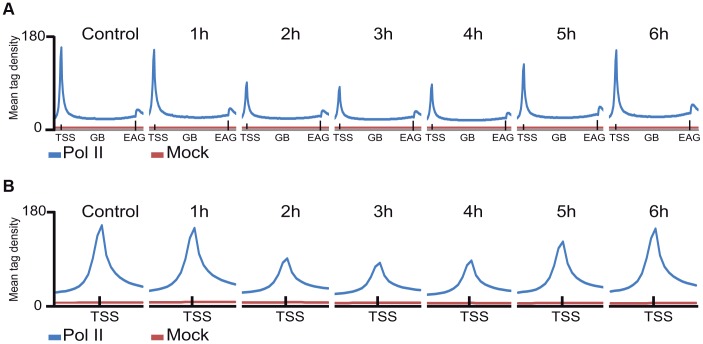
The average Pol II profile changes after UVB irradiation at all refseq genes. ChIP-seq was carried out using the anti-Pol II antibody (N-20) on human MCF7 cells 1–6 hours after UVB irradiation and on non-treated cells and the corresponding data sets were generated. Average Pol II profiles were calculated for every refseq gene from −1 kb region in front of every transcription start site (TSS), including the gene bodies (GBs), until +4 kb downstream from the 3′ end of annotated genes (EAGs) for all the time points (A) and −1 kb and +1 kb the around TSSs of every refseq gene (B). TSSs (in A and B) and EAGs (in A) are labeled. Pol II ChIP-seq signals are represented in blue and the mock ChIP-seq signals in red. The Y axis shows mean tag densities. Data sets obtained from irradiated cells harvested at the indicated time points in hour (h) after UVB irradiation together with the non-irradiated (Control) cells are represented.

Next, Pol II density profiles on the coding regions of all refseq genes were calculated for all datasets by using seqMINER tool [37]. To this end, average Pol II tag density values on each ORF, starting −1 kb upstream and ending +4 kb downstream from every refseq gene were calculated and compared. The non-irradiated sample resulted in the canonical Pol II occupancy profile showing a high, sharp peak centered around +50 bp relative to the TSS, a low density profile on the gene body (GB) and a higher broad peak profile downstream from the EAG (Figure 2A, [11]. Surprisingly, when we compared the six UVB treated samples to the non-treated control sample, by aligning the calculated mean Pol II profiles together on the same scale, we observed an unexpected genome-wide loss of Pol II signal around the TSS region of the refseq genes in the samples that were UVB treated and harvested 2, 3 or 4 hours following the treatment (Figure 2A and B). This observation suggests that there is a general signaling pathway that i) stimulates paused Pol IIs to leave their promoter paused position, and/or ii) inhibits the formation of new initiation complexes and/or iii) removes Pol II from its promoter proximal pausing position somewhere between 2 to 4 hours after UVB irradiation. Interestingly, in the UVB-treated samples that were left for 5 and 6 hours to recover before ChIP, Pol II occupancy at the TSSs of all refseq genes increased to the initial levels, suggesting that Pol II transcription has been restarted after TCR has been completed (Figure 2A and B).

**Figure 3:**
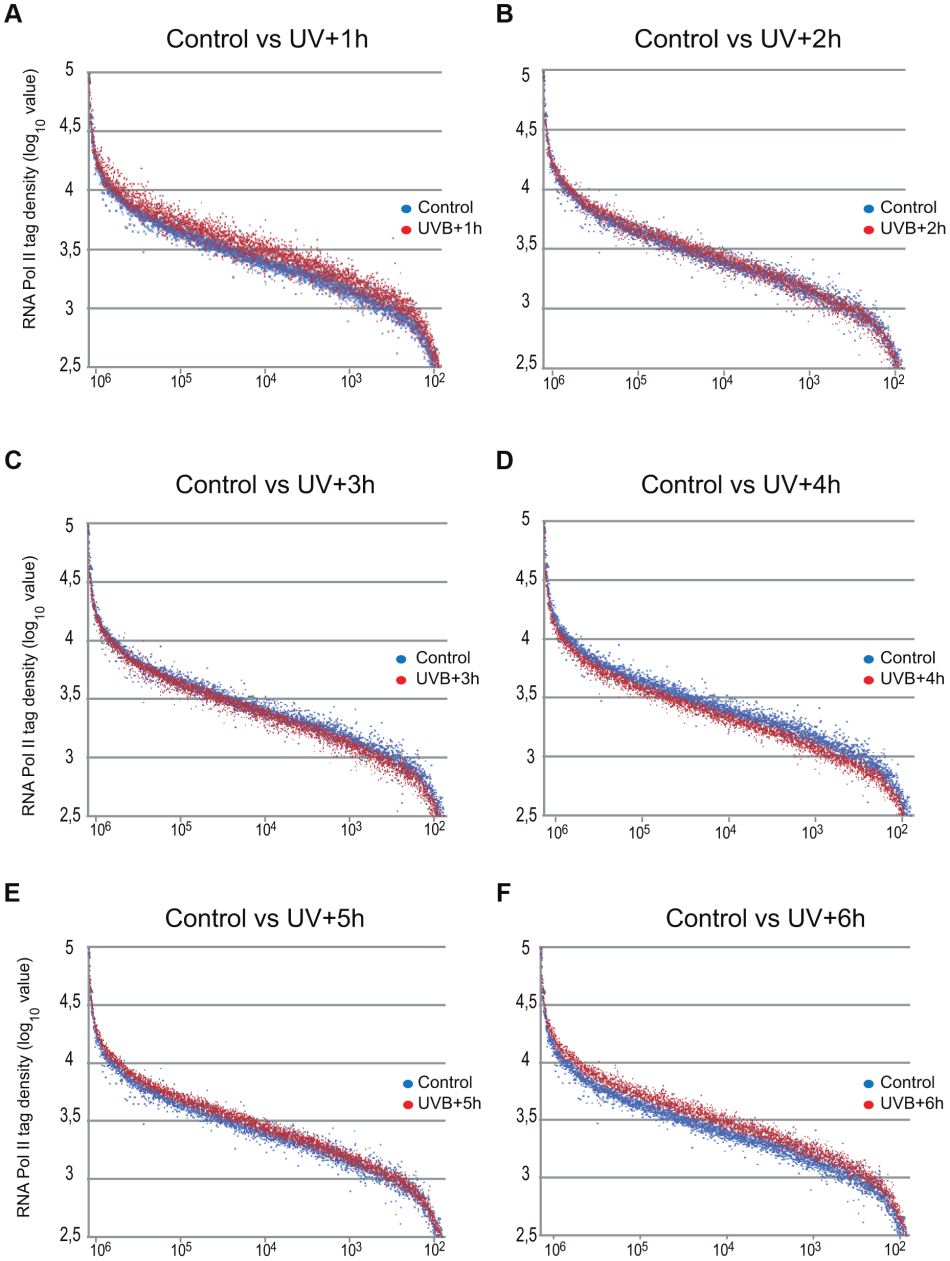
Global Pol II tag density changes dynamically in the gene body regions of all transcribed genes following UVB irradiation. Representation of Pol II read number changes obtained by ChIP-seq on MCF7 cell populations following UVB irradiation (as indicated) on the GB regions of the selected 4500 expressed genes when compared to that of the control sample. Pol II tag numbers were calculated on the GB (−100 bp from TSS until EAG) of the selected genes and represented in log 10 value on the y-axis. On the x axis, 4500 expressed genes are sorted based on their genomic lengths labeled in base pairs. Genes are classified from longest genes (from left) to shortest genes (at the right). Blue dots indicate tag numbers at genes in the control sample, and red dots indicate tag numbers at genes in samples harvested (A) 1 hour (h), (B) 2 h, (C) 3 h, (D) 4 h, (E) 5 h and (F) 6 h following UVB irradiation. See also Figure S2 for the total quantifications of the read numbers obtained in the different samples with the corresponding statistical tests.

As in the above Pol II binding analyses, when analyzing all refseq genes at gene bodies, we did not observe an obvious GW increase of Pol II occupancy, we next re-analyzed global Pol II tag density changes in the GB regions of all transcribed genes. For this first we selected 4500 expressed genes, from a recently published RNA-seq dataset for MCF-7 cell line [38] (for complete gene list see Table S2). These analyses clearly indicated i) a significant quick increase of Pol II tag density at all transcribed genes 1 hour following UVB irradiation; ii) followed by a gradual decrease of Pol II binding in GBs that sink under control levels at 3–4 hours following UVB, and iii) a novel global increase of Pol II signal that rises again above control levels at 5–6 hours following IVB irradiation (Figure 3 panels A–F). Next, we calculated the total Pol II reads on the gene body of the 4500 highly expressed genes in the 6 time point samples following UVB irradiation (Figure S2). In agreement with our genome wide analyses (Figure 3 panels A–F), we have found a statistically significant increase of Pol II global signal in the gene body regions of all the 4500 expressed genes in the 1-hour sample, a decrease at 3–4 hours and an novel increase at 5–6 hours following UVB irradiation. The increased Pol II occupancy values observed genome-wide at GBs 1 hour after UVB irradiation may represent the blocked Pol II complexes that are located at different lesions in the analyzed cells population. The decrease of Pol II tag density below control levels at 3–4 hours seems to reflect reduced transcription initiation (see below) and/or removal of Pol II from the transcribed genome. The novel increase in the GB regions at 5–6 following UVB irradiation may be responsible of the restarting of transcription (see Discussion).

### UVB induces a specific reorganization of Pol II occupancy on distinct regions of transcription units

**Figure 4:**
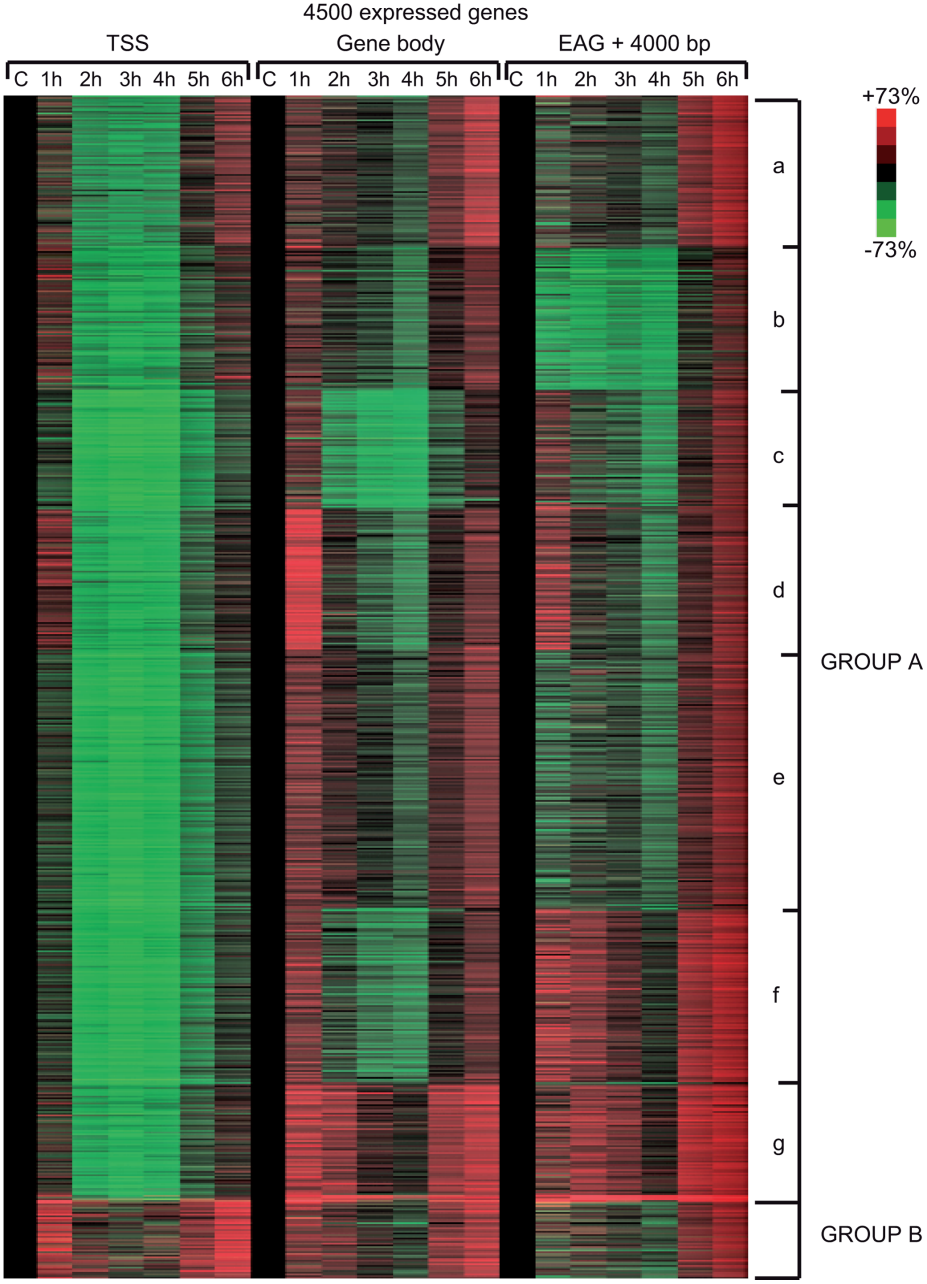
Different Pol II behavior patterns in time after UVB irradiation on 4500 expressed genes. From the ChIP-seq datasets heat maps were generated to follow Pol II behavior in time on the 4500 expressed genes at different genomic regions after UVB treatment in MCF7 cells. Tag numbers were calculated and visualized around the TSS (−/+300 bp), on the gene body (−100 bp from TSS until EAG) and downstream from EAG (from EAG to EAG+4 kb) of each gene. To be able to uniformly follow and represent the diverse Pol II occupancy changes on thousands of genes, the calculated Pol II reads at the different regions of different genes (as indicated) were converted into percentage values, where the control non-UVB treated values represent 100% reads (black color code) and the Pol II read alterations are either represented as % loss (green color) or as % gain (red color code). Each horizontal line represents one gene. The intensity of the color on the heatmap represents the magnitude of the Pol II tag number alteration (max −/+73%). Black color also refers to the lack of Pol II density change. In addition, genes were sorted into distinct groups and subgroups (Group Aa-Ag and Group B) based on the Pol II tag density values and patterns by using k-means clustering. Different time points following UVB irradiation are labeled at the top of the panels in hour (h). C = control, non-irradiated data set. For individual genes see Figure S3.

In order to carry out a more detailed investigation of the effect of UVB irradiation on the different phases of Pol II transcription, we calculated Pol II tag densities around TSSs (−/+300 bp), along the GBs of the genes (from TSS +100 bp to EAG) and downstream from EAG (from EAG to EAG +4 kb) regions of the 4500 expressed genes from the control data set and the six UVB irradiated samples (Figure 4). In addition, to identify genes with different Pol II behavior patterns we sorted the 4500 genes into clusters by using k-means clustering (Figure 4). With this method, by using the calculated Pol II reads, we sorted the genes into distinct groups based on Pol II occupancy pattern and density. During cluster and heat map generation to visualize Pol II density changes, values from all three regions (TSS, GB and downstream from EAG) were considered for the calculations including the 4500 expressed genes under control (c) conditions and at each of the 6 time points upon UVB irradiation (Figure 4).

From the heat maps it is visible that the generated clusters represent genes with distinct, unique Pol II transcription responses following UVB treatment, as we can observe well-defined differences in the changes of Pol II distribution at the different regions of the annotated genes. We found that the 4500 expressed genes can be sorted into two main groups, hereafter called A and B. Group A contains about 93% of the examined genes (Table S2) and shows in contrast to group B dramatic Pol II signal loss from the promoters between 2 and 4 hours post UV irradiation (Figure 4 and Figure S3). While on the gene promoters of group A an almost uniform Pol II signal loss can be observed between 2 and 4 hours after UVB irradiation, this group can be further subdivided into additional subgroups, depending on different Pol II behavior patterns observed mainly in the GB and/or the EAG+4000 regions (see Aa-Ag in Figure 4 and Figure S3). Moreover, to analyze and find potential differential gene function categories between the detected distinct Pol II behavior patterns on genes belonging to the different groups and subgroups, we carried out Gene Ontology (GO) analyses on the identified categories of genes (Table 1, carried out with D.A.V.I.D.; and Table S1, carried out with MANTEIA).

**Table 1:** Results of gene ontology analyses carried out using the D.A.V.I.D. software to identify differential gene-function categories for the detected gene groups with distinct Pol II behavior patterns from Figure 4.

Group	GO term (D.A.V.I.D)	p-value
Aa	non-membrane-bound organelle	7,40E-05
	RNA splicing	7,06E-05
	mRNA processing	1,10E-04
Ab	negative regulation of macromolecule metabolic process	3,54E-11
	negative regulation of gene expression	7,81E-08
	macromolecule catabolic process	3,79E-06
Ac	ribonucleoprotein complex	1,54E-25
	translation	1,38E-18
	ubl conjugation	6,79E-14
Ad	nuclear mRNA splicing, via spliceosome	9,22E-15
	intracellular organelle lumen	8,46E-15
	mRNA processing	1,54E-13
Ae	nucleotide binding	7,14E-13
	mitochondrial matrix	3,88E-11
	ATP binding	1,51E-07
Af	structural constituent of ribosome	4,71E-27
	mitochondrion	3,27E-18
	translational elongation	1,42E-16
Ag	intracellular organelle lumen	6,33E-11
	response to radiation	2,67E-04
	negative regulation of macromolecule metabolic process	1,34E-04
B	DNA damage response, signal transduction by p53 class mediator	6,30E-07
	DNA damage response, signal transduction	4,60E-06
	transcription regulator activity	7,00E-04

Interestingly, in many sub-clusters Pol II occupancy increased at 1 hour and then again at 5 and 6 hours following UVB treatment at distinct regions of the transcription units (Figure 4 and Figure S3). Compared to the other patterns, genes in subgroup Aa have a strong increase of Pol II signal at 6 h on TSS, GB and EAG+4000 regions, while they have a somewhat weaker decrease of Pol II signal at their TSS regions between 2 and 4 hours than subgroups Ac-Ag (Figure 4 and Figure S3) (for gene lists see Table S2). Genes in the Aa subgroup belong predominantly to ‘RNA splicing’ and ‘mRNA processing’ GO categories (Table 1, as defined by DAVID; and Table S1 as defined by MANTEIA; [39], [40], see Materials and Methods). Genes in subgroup Ab have also a somewhat weaker decrease of Pol II signal at their TSS regions between 2 and 4 hours when compared to the Ac-Ag subgroups, but have a strong decrease of Pol II signal in the EAG+4000 region between 1 and 4 hours, suggesting that on these genes Pol II is rapidly terminating and/or removed from these 3′ regions. Interestingly, this subgroup contains a number of genes that belong to the ‘negative regulation of macromolecule metabolic process’ and ‘negative regulation of gene expression’ GO categories (Table 1). In genes belonging to subgroup Ac, in addition to the strong Pol II disappearance at the TSS region, Pol II signal decreases very strongly in the GB region between 2 and 4 hours, while this decrease is not apparent in the EAG+4000 region. Importantly, this subgroup contains mainly genes involved in ‘ribonucleoprotein complex formation’, ‘regulation of translation elongation and termination’ GO categories (Table 1). Genes belonging to the subgroup Ad amongst other functions play a role in ‘mRNA metabolic process’ and have a very strong Pol II increase in the GB one hour after UVB irradiation suggesting that the gene products are very quickly required after UVB irradiation. Genes in subgroup Ae have a very strong Pol II decrease at their promoters, but relatively modest changes in their GBs and EAG+4000 regions. These genes, amongst other functions, fall in the ‘nucleotide and ATP-binding’ GO categories. Genes belonging to the subgroup Af have a very strong increase of Pol II density following irradiation at 1 hour in the GB, and at 1–2 and 5–6 hour at the AEG+4000 region, suggesting that these genes may be stimulated during the first hour after UV irradiation and after that Pol II accumulates downstream from the genes. Genes belonging in the ‘structural constituent of ribosome’ and ‘translation elongation’ GO categories are overrepresented in this subgroup. The subgroup Ag consists of genes, which show Pol II signal loss from their promoters, but show a quick and almost constant increase of Pol II enrichment on GB and EAG+4000 regions. Interestingly, amongst other transcription units, genes in the ‘response to radiation’ or ‘response to UV’ GO categories (Table 1 and Table S1) are overrepresented in this subgroup suggesting that these genes are heavily transcribed, but without having a paused Pol II at their promoters (Figure 4 and Figure S3). Note that in the above clusters we did not observe any correlation between gene length and Pol II behavior following UVB irradiation.

In contrast to group A, the relatively small set of genes in group B (containing 322 genes, Table S2) is characterized by no general loss of Pol II signal from their promoter regions (Figure 4 and Figure S3). In addition 1 and 5–6 hour after irradiation a strong increase of Pol II occupancy can be observed at the promoters of these genes. Moreover, in general in these genes a significant increase of Pol II signal through the entire transcription unit can be observed after irradiation. Interestingly, genes belonging to group B are overrepresented (p-values 6E-07-4E-06) in the ‘DNA damage response’ and ‘DNA damage response, signal transduction by p53 class mediator’ GO categories, further validating the relevance of our Pol II ChIP assays and bioinformatics classifications.

These results together suggest that a general negative regulation of Pol II transcription exist in response to UVB irradiation. Moreover, on distinct regions of the transcription units the presence of Pol II is differentially regulated following UVB irradiation, probably also depending on the function of the genes. Nevertheless, the response to UVB irradiation can mainly be broken down in two categories of genes: those where Pol II presence at the promoters is down regulated after irradiation (group A, about 93% of the genes) and genes, out of which many regulate DNA damage response, signal transduction by p53 and apoptosis, where Pol II presence is increased in the TSS, GB and slightly at EAG+4000 regions (group B, less than 10% of the expressed genes). Note however that most of the NER factors are abundant in the nucleus [41] explaining why certain NER genes can be found in group Ag instead of group B.

### Many annotated repair genes show Pol II increase in response to UVB treatment

**Figure 5:**
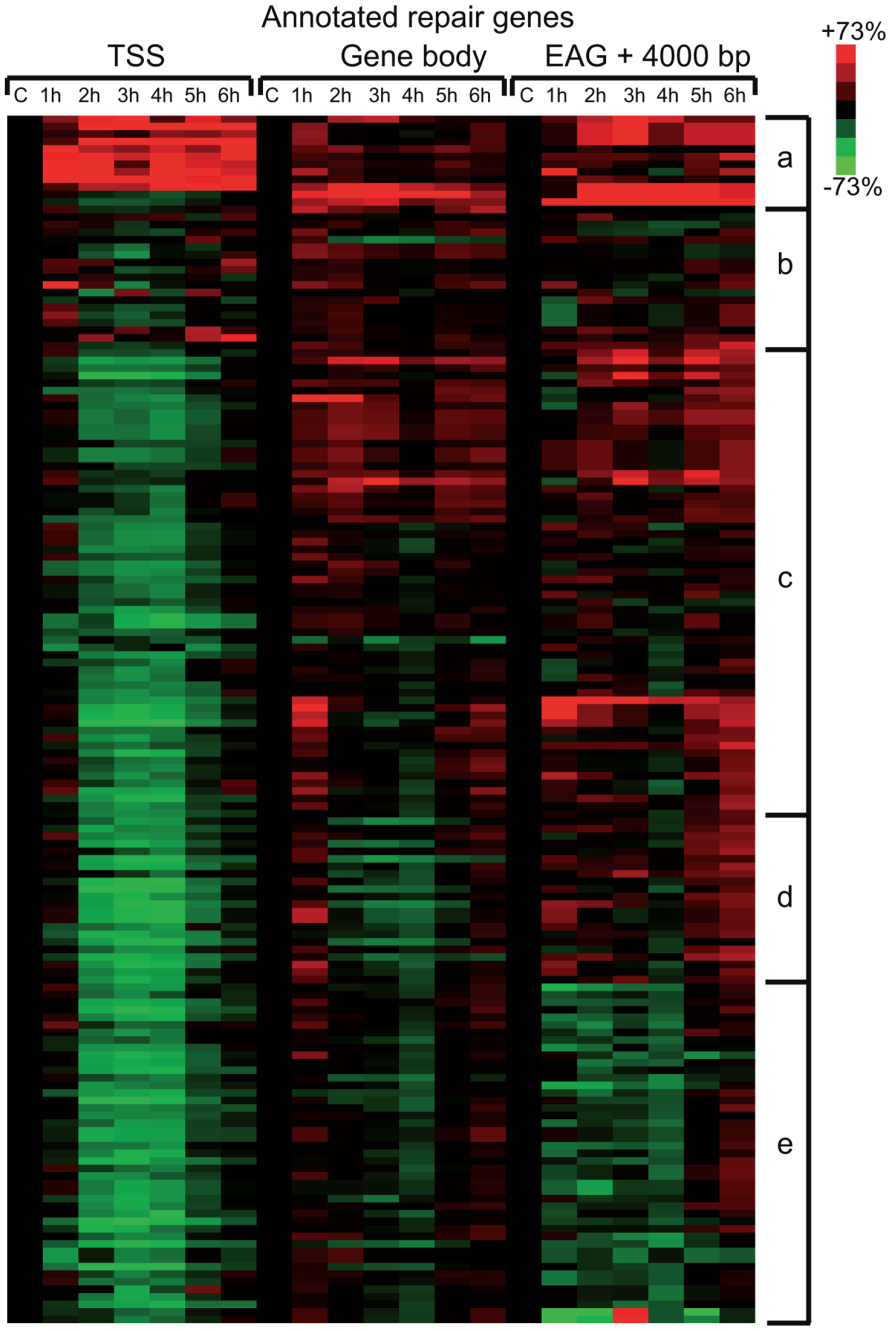
Pol II behavior on annotated DNA damage repair and UV responsive genes after UVB irradiation. Pol II reads were calculated around the TSS (+/−300 bp), on the gene body (−100 bp from TSS until EAG) and downstream from EAG (from EAG to EAG +4 kb) for well characterized DNA repair and UV responsive genes (from the KEGG database) from each ChIP-seq dataset. From the collected values heat map was generated, and genes are organized into groups based on similar Pol II behavior by using k-means clustering. Pol II occupancy changes are represented as in Figure 4. Red color shows increased; green color shows decreased Pol II tag numbers in percentages compared to data obtained from the control non-UV irradiated cells (black color). Black color also refers to no change in Pol II density. The figure is labeled similarly as Figure 4.

In the above detailed global analysis of Pol II behavior on expressed genes upon UVB treatment we have observed that many genes in group B, belonging to the GO categories ‘response to UV’ and ‘DNA damage response’, have increased Pol II occupancy throughout their whole transcription unit. Thus, we analyzed Pol II distribution at annotated repair and UV-responsive genes existing in the KEGG database [42], which may have been missed in the above analyses because they are not expressed under ‘normal’ conditions. We clustered the 164 characterized and annotated repair genes according to their Pol II occupancy and created heat maps as above (Figure 5) (for gene lists see Table S2). These analyses indicate that in about half of the annotated repair genes Pol II signals increase in all of the three regions or in only part of them. We observe the following categories: a) Pol II tag density increases everywhere in the transcription unit with a either a strong increase at the TSS or with a strong increase only in the GB and EAG+4000 regions at almost all the analyzed time points, b) Pol II occupancy slightly increases at all three regions of the transcription units following UVB treatment, c) while Pol II occupancy decreases at the TSS regions, its increase is more restricted to the GB and EAG+4000 regions, d) Pol II is increasing only at the EAG+4000 region, e) Pol II occupancy does not increase, but rather decreases at all three analyzed regions (Figure 5). Interestingly, in a) and b) categories there are several genes, such as *DNA ligase IV (ATP-dependent)*, *cyclin D2, p21* (also called *cyclin-dependent kinase inhibitor 1A*), *GADD45A* and *GADD45B*, and *TP53AIP1, SESN2, FAS, BBC3*, which are regulators of repair, cell growth or survival pathways further validating the biological significance of the present Pol II occupancy study.

### Pol II and PIC subunits are not degraded following the sublethal UVB irradiation

**Figure 6:**
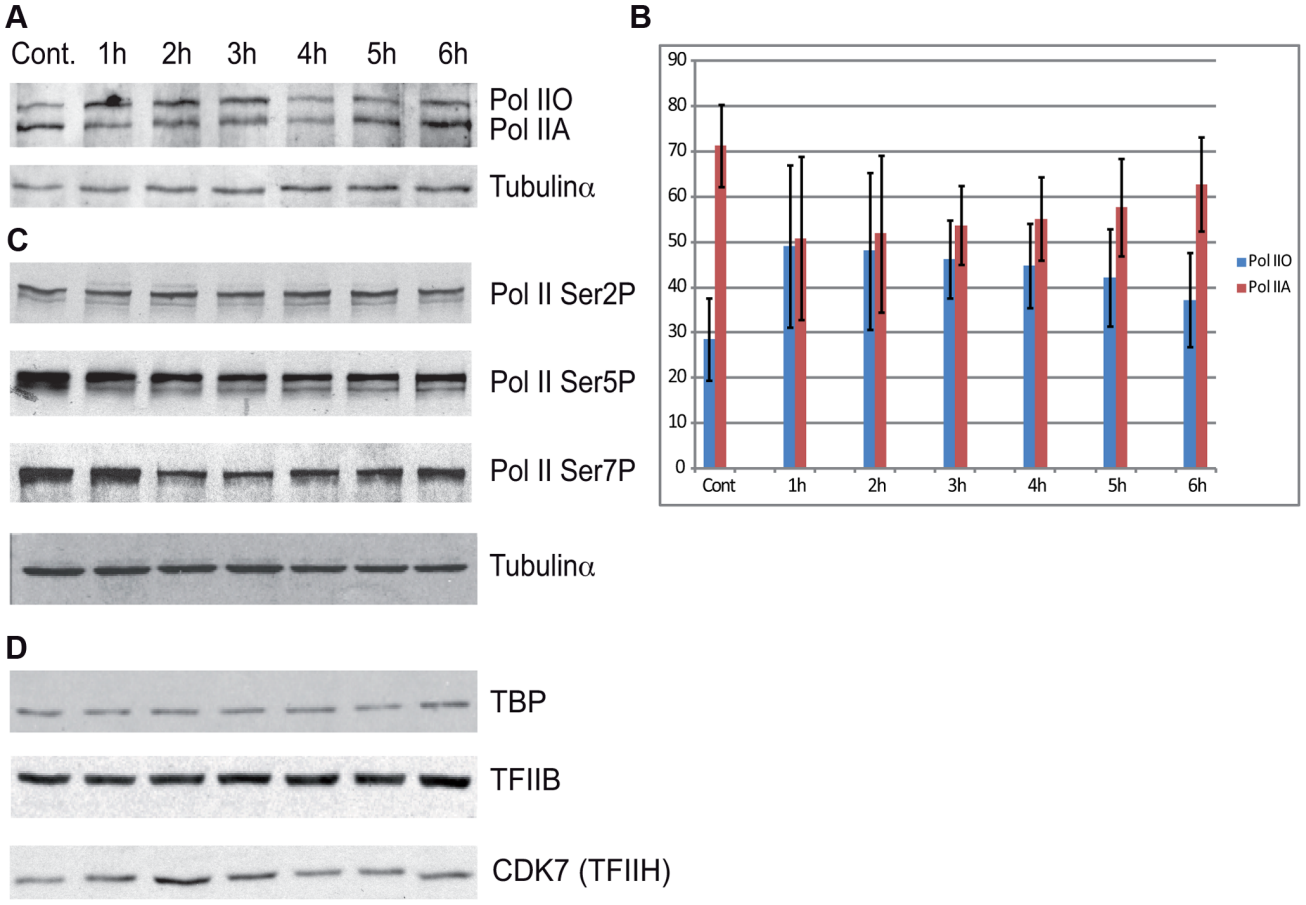
The protein level of Pol II and GTF subunits does not change after UVB treatment. (A) Western blot assays were carried out to measure the levels of total Pol II at the indicated time points after UVB treatment by using the N-20 antibody. (B) The different IIA and IIO forms of Pol II were densitometrically quantified in each time points from four independent experiments and are represented in a bar chart, where red bars indicate Pol IIA and the blue bars indicate Pol IIO forms. In each lane Pol IIA+IIO signals are taken as 100%. Error bars represent +/− standard deviation of the four independent experiments. (C) The phosphorylation levels of Pol II Rpb1-CTD Ser2, Ser5 and Ser7 were analyzed by western blot by using the indicated antibodies, respectively. (D) The levels of the indicted GTF subunits such as TFIIB, TBP and TFIIH/CDK7 were also tested in the above prepared extracts, as indicated. Tubulin-α was used as a loading control.

To evaluate whether the above observed massive Pol II promoter clearance at 2–4 hour time points in Group A (Figure 4) is due to degradation of Pol II or other PIC subunits, we prepared cell extracts from non-irradiated cells and cells 1–6 hours following UVB irradiation and tested the presence of the indicated PIC subunits by western blot assay (Figure 6). The N-20 antibody recognizes Pol IIA and Pol IIO forms of Rpb1, which have been suggested to correspond to hypo- (IIA) and hyperphosphorylated (IIO) carboxy-terminal repeat domains (CTDs), respectively [43], [44]. Note, however, that these two very discrete forms of Pol II may be due to other more complex modifications as well. Importantly, our immunoblot assays indicated no detectable degradation of Rpb1 in several independent experiments (Figure 6A and B, and data not shown). Moreover, examination of the distributions of the differentially migrating forms of Pol II revealed that between 1 and 3 hours following UVB irradiation the normal balance between Pol IIA and Pol IIO forms (70/30%, respectively) is shifted towards the IIO form (50/50%), and that by 6 hours after UVB irradiation the Pol IIA and Pol IIO balance is again close to the normal non-irradiated ratio (65/35%). Thus, our results seem to be in good agreement with previous studies showing that upon strong doses of UVC caused DNA damage Pol II is hyperphosphorylated and/or the Pol IIO forms becomes dominant [45], [46]. This could also explain the observed promoter clearance, as Pol II needs to be hypophosphorylated to form the PIC.

Additionally we tested the level of the phosphorylation of the CTD of Rpb1 using antibodies that recognize different and specifically phosphorylated forms of the CTD heptapeptide repeats (Figure 6C). In this assay Ser2-P of Pol II CTD does not show any significant alterations upon UVB irradiation. In contrast, Ser5-P signal of Pol II CTD decreased immediately 1 h after UVB irradiation and this lower level seemed to be maintained during 6 hours following irradiation with a hint of recovery at the last time point. In addition, we detected a decrease in the level of Ser7-P signal upon UVB treatment between 2–4 hours; and a progressive reappearance of the Ser7-P from the 5 h time point following irradiation. This signal seems to follow the behavior of Pol II occupancy on the majority of the genes in Group A and is in good agreement with the finding that Ser7-P CTD may be a marker of transcription from expressed genes [44], [47]. Importantly, none of the tested Pol II signals indicate the degradation a Pol II Rpb1 and/or its CTD following the 55 J/m2 dose of UVB irradiation.

To test whether the degradation of additional PIC subunits would be responsible for the important Pol II clearance from the promoters upon UVB irradiation, we tested the global protein levels of TBP, TFIIB and the kinase subunit of TFIIH, CDK7, which is known to phosphorylate the CTD of Pol II [48] [44]. Our analyses do not show any significant changes in the levels of the tested proteins (Figure 6D). These results suggest that the loss of Pol II signal from promoters after UVB irradiation is not due to a general degradation of PIC components is the nucleus.

### Differential Pol II behavior at individual promoters

**Figure 7:**
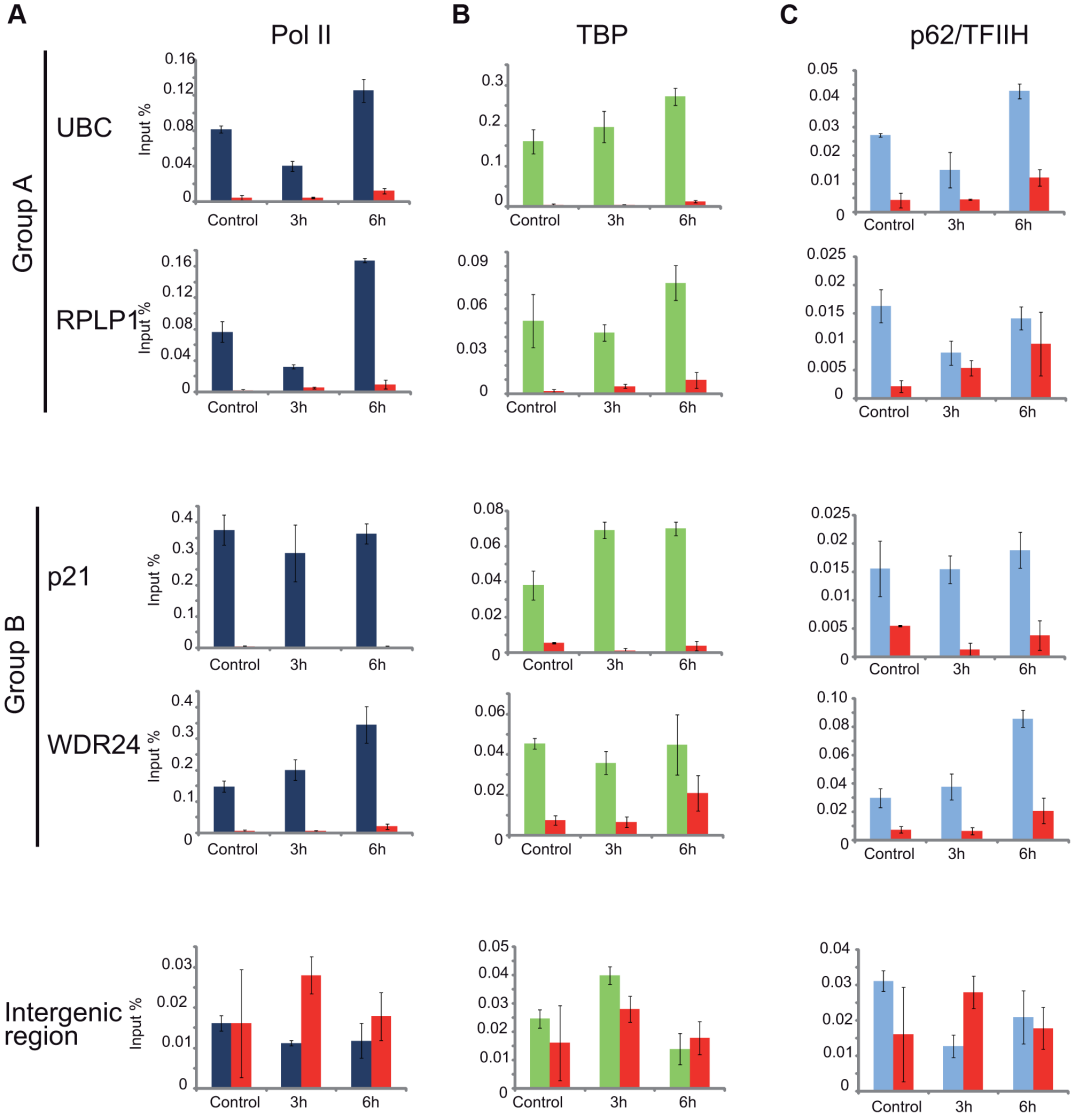
ChIP-qPCR validation of Pol II and PIC subunit behavior on promoters of group A and B genes. ChIP was carried out 3 and 6-treated cells and analyzed by quantitative (q)-PCRs to monitor Pol II (dark blue bars) (in A), TBP (green bars) (in B), p62 (light blue bars) (in C) occupancy on the promoters of genes selected from group A (*ubc, rplp1*), group B (*p21, wdr24*) and on a negative control (*intergenic*) region, as indicated. Control ChIP (NoAb, red bars) was carried out with Sepharose G beads only. The occupancy values at the promoters are represented in input %. Error bars represent +/− standard deviations.

As the general disappearance of Pol II signal from the promoters of Group A genes following UVB irradiation does not seem to be due to Pol II or other PIC component degradation, next we set out to validate the bioinformatically detected different Pol II behavior categories at the promoters (Pol II clearance at group A and stable or increasing Pol II signal at group B). To this end we used anti-Pol II ChIP coupled qPCR detection on non-treated samples and on samples incubated for 3 h and 6 h after 55 J/m^2^ UVB irradiation. Pol II signals were quantified on the promoters of two randomly selected genes from group A (*rplp1 and ubc*) and B (*p21 and wdr24*). Negative/mock control ChIP was carried out with Sepharose G beads alone (NoAb), and for an additional control, oligonucleotides were designed to target an intergenic region, where no Pol II binding is expected (Figure 7A).

The ChIP-qPCR experiment confirmed the different Pol II behavior patterns at the promoters of the selected genes. At the promoters of genes from group A we observed a decreased Pol II occupancy in the sample that was harvested 3 hours following UVB irradiation when compared to the control (Figure 7A). As expected from the ChIP-Seq and bioinformatics results (Figure 4), both genes from group A show an increased Pol II occupancy on their promoter region in the sample that was harvested 6 hours following UVB irradiation (Figure 7A). Genes from group B show either no decrease (*p21*) or increased (*wdr24*) Pol II enrichment at the promoters at 3 hours after UVB treatment compared to the control. In the case of *wdr24* gene Pol II enrichment increases up to 6 hours post UVB treatment. These ChIP-qPCR validations are in good agreement with our ChIP-seq, bioinformatics and IF results.

### TFIIH, but not TBP, follows the behavior of Pol II at promoters of group A genes upon UVB treatment

Next, to better understand the mechanisms that may regulate the opposite Pol II behavior on gene promoters belonging to either group A or B, we investigated whether certain PIC subunit enrichments would also be affected upon UVB irradiation. To this end we carried out ChIP-qPCR detection using antibodies against subunits of two GTFs, the TATA-box binding protein (TBP) a subunit of TFIID, and p62, a subunit of the TFIIH (Figure 7B and C). Surprisingly, TBP showed relatively stable or even increasing occupancy patterns at every tested gene from group A and B and its binding seemed to be resistant to the events that cause Pol II dissociation from the promoter (Figure 7B). In contrast, the p62 subunit of TFIIH followed the same behavior as Pol II. The detectability of p62 by ChIP decreased on the promoters of genes belonging to group A, but it was stable on genes from group B 3 hours following UVB irradiation (Figure 7C). At 6 hours following UVB irradiation, p62 presence at promoters recovered to the non-irradiated control levels or even higher. Thus, it seems that while TBP-containing partial PICs, or reinitiation complexes stay at the group A promoters at 3 hours following UVB irradiation, TFIIH disappears from promoters together with Pol II. These results suggest that on group A genes upon UVB irradiation transcription might be blocked to prevent PIC formation that in return would provide “free” TFIIH and time for TCR (see Discussion).

### In the absence of CSB UVB does not inhibit Pol II and TFIIH binding at promoters of group A genes

**Figure 8:**
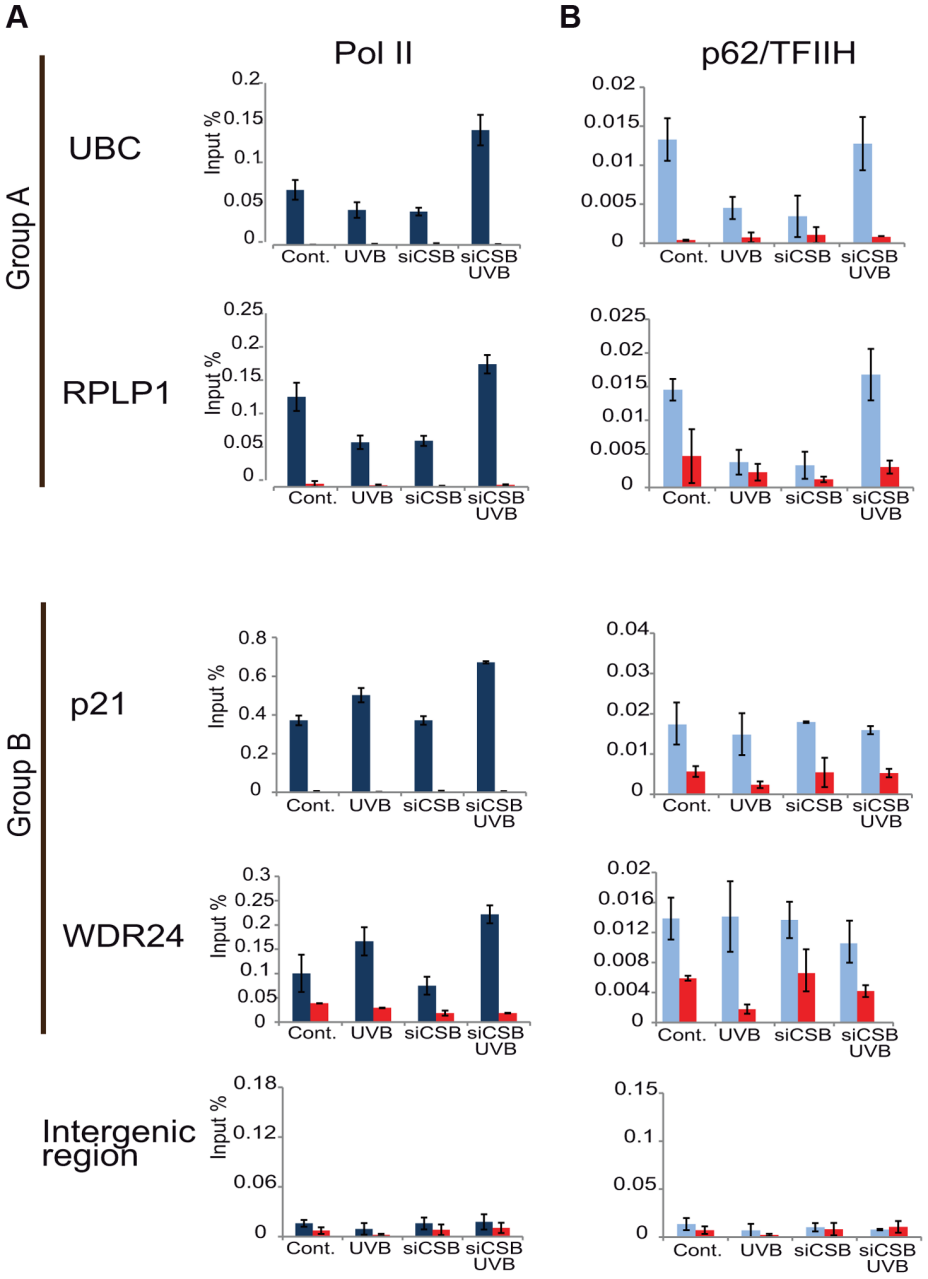
In the absence of CSB UVB does not inhibit Pol II and TFIIH binding at promoters of group A genes. MCF7 cells were transfected with either scrambled siRNA or siRNAs targeting CSB (ERCC6) (see also Figure S4), 72 hours following siRNA transfection cells were treated with UVB, or not. ChIP was carried out 3 hours following UVB treatment of MCF7 cells as well as on non-treated cells. Q-PCRs were carried out to monitor Pol II (dark blue bars) (in A), and p62 (light blue bars) (in B) occupancy on the promoters of genes selected from group A (*ubc, rplp1*), group B (*p21, wdr24*) and on a negative control (*intergenic*) region in the presence or absence of CSB, as indicated. Control ChIPs (NoAb, red bars) was carried out with Sepharose G beads only. The occupancy values at the promoters are represented in input %. Error bars represent +/− standard deviations in two biological replicates.

CSB is known to trigger the recruitment of NER factors, including TFIIH, to UV-induced DNA lesions to carry out the repair process (see Introduction). Thus, to test our above hypothesis concerning the sequestration of TFIIH by the TC-NER pathway away from PIC formation, we have knocked down CSB expression by using siRNA transfection in MCF7 cells (Figure S4), and have tested whether under the CSB knock-down condition Pol II and TFIIH recruitment would still be inhibited to group A promoters by UVB (Figure 8A and B). In good agreement with our hypothesis, when cells were treated with siRNA against CSB, UVB did not reduce either Pol II or p62/TFIIH recruitment to the promoters of the tested group A genes, whereas at group B gene promoters siCSB had no significant effect on either Pol II or TFIIH recruitment induced by UVB (Figure 8A and B). These experiments further suggest that CSB and the NER pathway participate in employing the pool of TFIIH that in turn would not be available for participating in PIC formation (see also Discussion).

## Discussion

### Sublethal dose of UVB generates persistent CPD lesions throughout the genome for more than 24 hours, but transcription restarts at 6 hours

In the past decades a lot of efforts have been devoted to understand how Pol II transcription is recovered after genotoxic stress, such as UV irradiation. However, our knowledge about Pol II transcription inhibition during TCR and the subsequent recovery of RNA synthesis is based only on a handful of model genes. Note that most of the previous studies that investigated the link between repair and transcription used much higher, often lethal, UVC doses [49]. To analyze Pol II behavior following UV irradiation, we carried out seven parallel ChIP-seq experiments following sublethal UVB treatment of human MCF7 cells to track DNA-bound RNA Pol II complexes genome-wide over time.

Importantly, the sublethal 55 J/m^2^ dose of UVB irradiation used had a wide effect on the genome of MCF7 cells, as it induced CPD lesions genome-wide, which persisted up to, or even longer than 24 hours (Figure S1). Despite the presence of these lesions (and probably other type of lesions) in the genome, the transcription and initiation capability of Pol II got restored, and/or even stimulated, 5–6 hours post UVB irradiation in the cells (Figure 1–4) suggesting that CPDs have been repaired in the transcribed genome, but not yet in the intergenic regions. Thus, our observations are in good agreement with previous studies [21], [22], and reflect the differences in speed and efficiencies between TCR and GGR, and show that the genome wide UV-induced negative regulation is very dynamically relieved after TCR (see also below).

### At the majority of transcribed genes UVB induces a rapid regulatory mechanism that removes promoter-paused Pol II and/or blocks formation of new PICs

Our results show that on about 93% of the promoters of expressed genes Pol II accumulates at the TSSs during the first hour after irradiation, Pol II occupancy is seriously reduced 2–4 hours following UVB irradiation, and that the presence of Pol II is restored to “normal”, or even sometimes “overshoot” levels 5–6 hours after irradiation (Figure 4 and Figure S3). These results seem to be in apparent contradiction with the observed decrease of nascent transcripts (as measured by incorporation of 5-fluorouridine) in the first hour post irradiation, followed by a constant increase between 3 and 6 hour after UVB (Figure 1). Note however, that incorporation of nucleotides labels all transcripts at a single cell level produced by the three RNA polymerases, including many non-coding transcripts, abortive transcripts produced during promoter clearance, and also short upstream antisense transcription start site associated RNAs (TSSa-RNAs) and many others [36], whereas Pol II ChIP-seq visualizes crosslinkable Pol II molecules on the genome in a large cell population. Thus, the two methods are complementary and together suggest that following UVB stress in MCF7 cells there is a very rapid global reduction in transcript production in general that is reassumed from 3 hours onwards after UVB irradiation, while the ChIP-able genome-wide binding of Pol II reaches a minimal level between 2 and 4 hours and is returning to normal or even higher levels 5–6 hours after irradiation.

The reduction of the Pol II signal at promoters can be the result of several molecular events acting either independently or in combination: i) Pol II assembled in a PIC and pausing at or close to the TSSs runs in the gene body, ii) new PIC formation does not occur, or iii) existing PICs containing paused Pol II are actively removed from TSSs. Nevertheless, our observations strongly suggest the existence of a rapid negative regulatory mechanism, which seems to act on almost all expressed gene promoters during TCR to either remove existing PICs, containing paused Pol IIs, and/or prevent Pol II to enter in initiation, or re-initiation, complexes at promoters, when transcription-blocking lesions may still be present on the ORFs. The fact that promoter-proximal pausing of Pol II at group A genes 2–4 h after UVB irradiation decreases everywhere (Figure S3 and Figure 4), independently of the heights of the Pol II peaks (or Pol II residency time), suggests that the negative regulation causing the genome-wide Pol II clearance on promoters influences “strong” or “weak” promoter-proximal pausing of Pol II the same way.

In agreement, an active global transcriptional repression process, rather than a physical blocking of transcription, has already been suggested [32]; and refs therein). Such a negative regulation would operate at several levels on most of the transcribed genes. Pol II molecules, which have been loaded on the promoter and/or the gene body at the moment of the UVB irradiation would quickly run into the DNA lesions, stop and signal the road blocks (Figure 3). This signalization would in response have dual effects: i) induce the recruitment of the TCR-NER to the lesions and ii) trigger the inhibition of transcription at most transcribed genes. In agreement, it has been suggested that transcription arrest and DNA repair can be separated [50]. Along these lines, it has also been shown that ATF3 expression is induced following UVC irradiation, ATF3 is recruited to its binding site located in the vicinity of a subset of promoters where it represses transcription of its target genes [50]. Nevertheless, this mechanism would only partially explain the genome-wide observed Pol II clearance at TSSs described in the present study.

It is crucial for the cell that DNA lesions are repaired in the different transcription units with equal efficiencies, in order to avoid that short genes with low damage probability are repaired and transcribed quicker than long genes with a high likelihood of carrying many UV-induced CPD lesions. We do not know whether this negative “preventive” transcription regulatory mechanism can operate at several levels separately (i.e. remove existing PICs containing paused Pol II and/or block Pol II incorporation in new PICs) or whether it would inhibit both mechanisms by blocking the function of the same factor for example. Interestingly, it has been observed that the loss of de novo RNA synthesis occurs only at the UV-irradiated area of the nucleus [51], suggesting that the transcription inhibition does not spread to long distances in the nucleus.

The transcription factor TFIIH was shown to play an essential role in both transcription initiation and DNA repair [20], [52]. As on TSSs of genes of group A, Pol II and TFIIH are reduced comparatively 3 hours following irradiation (Figure 7), it is conceivable that after UVB irradiation TFIIH is sequestered away by the TC-NER machinery (including CSB) from both existing and newly forming PICs by the repair machinery. This in return would help to open the DNA around the lesion and thereby allow the excision of the damaged oligonucleotide and its replacement by a new DNA fragment genome-wide [20]. Our results showing that following the depletion of CSB, one of the first factors of the TC-NER machinery that recognizes the DNA lesions, neither Pol II nor TFIIH binding is reduced to the promoter of group A genes after UVB irradiation (Figure 8), further corroborates this model. Note however, that CSB was reported to play a role in the UVC-dependent degradation of the large subunit of Pol II [46], making it possible that the depletion of CSB could also increase Pol II stability in our assay.

In line with the sequestration model by the TC-NER machinery, earlier work showed that TFIIH engagement in transcription initiation takes only a few seconds, whereas when it engages in NER it takes minutes for every NER event, which may explain why the pool of TFIIH is quickly depleted and shifted towards repair [53], [54]. In theory, as TFIIH is present in the PICs or re-initiation complexes of the transcribed genes, the promoter-bound TFIIH molecules would be easier/quicker to recruit to the neighboring DNA lesions occurring in the same transcribed units than “freely” diffusing TFIIH molecules present in the nucleoplasm, in agreement with the observation of [51]. Moreover, it has been demonstrated that very quickly after UV irradiation TFIIH changes its subunit composition, by loosing its kinase sub-module (called CAK) needed for transcription initiation, and the core TFIIH (that does not contain CAK) subsequently associates with DNA repair factors and gets recruited to the sites of NER [55]. Such an UV-induced TFIIH “promoter deprivation” and dissociation mechanism (possibly from both existing and newly forming PICs) would explain why the presence of Pol II would decrease at transcribed promoters and why the repair machinery would repair the transcribed regions quicker than other genomic regions. Such a regulatory step inhibiting PIC formation and the consequent Pol II molecules running in the gene bodies would be very important to avoid the accumulation of Pol II complexes around the DNA lesion sites. Our model would also predict that transcription units, which are silent, or intergenic regions without Pol II transcription, would be repaired slower.

The detected synchronized Pol II reappearance on the TSSs of most of the transcribed genes 5–6 hours after UVB irradiation suggests that transcription-coupled repair is largely completed by this time in MCF7 cells; TFIIH complexes are released from the repair sites and again available for new PIC formation on the transcribed genes. Moreover, our results investigating the presence of TBP, a DNA-binding component of TFIID, showed that the presence of TBP at the TSSs of genes is not changing dramatically over time after UVB irradiation. This observation suggests that on the transcribed genes during UVB irradiation, and following the potential TFIIH sequestration by the repair machinery, a minimal TFIID/TBP-containing re-initiation complex may stay bookmarking the TSSs where transcription has to be resumed once TCR is completed.

### Pol II behavior is uniform on promoters of group A genes, but diverse on the GB and downstream from EAG regions

While the Pol II clearance and recovery seems to be synchronized at the promoters of group A genes, we found very diverse patterns of Pol II behavior on the GB and on the EAG+4000 regions of the expressed genes during recovery after UVB irradiation (Figure 4). Based on our GO analyses we speculate that the main reason for these characteristic Pol II occupancy differences amongst the distinct gene clusters may be due to their function in diverse cellular process. Alternatively or in addition, the structure or the function of a given gene product may also play a role in the differential Pol II presence in the transcription units. For example the comparison of Pol II patterns and the GO results of group Ac (GO terms: translational elongation and termination) and group Ag (GO terms: response to UV) clearly show that the genes in group Ag must be repaired and restored very fast as they might have important role in the recovery of normal homeostasis after UV stress.

Another common characteristic exists on the GBs of group A transcription units, namely that in most of the cases there is an increase of Pol II occupancy at 1 hour after irradiation on the GBs of these genes, which show Pol II clearance from their promoters (Figure 2 and 3). The increased Pol II signal in the GBs (Figure 3) may represent a synchronized “last” round of transcription that may be required to detect and signal the DNA lesions to start TCR.

Moreover, Pol II changes on the GBs and EAG+4000 regions did not always parallel the homogenous decrease in Pol II binding at the promoters of group A genes following UVB irradiation. This suggests that the UVB-induced global negative regulatory mechanisms affecting Pol II transcription initiation, elongation and termination may be distinct and are not always interconnected.

### At a small subset of transcribed genes the UVB-induced negative regulatory mechanism does not operate

We also detected a set of genes, which show no decreased Pol II presence at their promoter regions, but rather a general increase of Pol II density throughout their three analyzed gene regions after UVB irradiation (Figure 4). Genes belonging in this group (B) fall often in the GO categories annotated as DNA damage response, signal transduction and apoptotic processes. A separate search in repair-associated genes in the KEGG database also showed that a portion of genes in this category behave as genes in our group B. Many of these genes seem to be key genes regulating repair, cell cycle, apoptosis and stress responses. Thus, these genes with increase Pol II signals at their TSSs and other regions should have a particularity that differentiates them from the majority of the expressed genes in group A, and it is tempting to speculate that group B are somehow protected from the above described transcription inhibition mechanism. To overcome the negative regulation at the promoters of group B genes, several scenarios are possible: i) these genes may get repaired much faster than the genes in group A and at the 1 hour time point they are transcribing again; or ii) TFIIH is present, but indispensable for transcription initiation on these genes, and thus, the TFIIH sequestration mechanism does not influence the transcription of these genes; or iii) these promoters would have specific requirements for transcription initiation, involving slightly different, non-canonical initiation machinery that could for instance involve specific stress-related transcription factors and a special form of TFIIH. As TFIIH independent transcription initiation has already been reported [56], [57], [58], [59] it is conceivable that even if TFIIH was present in the PICs of the group B promoters, its sequestration by CSB and the repair machinery would not block transcription initiation on these genes (see also Figure 8). The above possibilities alone or in combination may ensure rapid clearance of the transcribed strand from damage and subsequent rapid transcription because of the need of the cell to use the encoded proteins.

It is known that p53 can regulate a subset of genes under compromised physiological conditions, when for example DNA is damaged. It is has been suggested using the *p21/cdkn1a* model gene that p53 uses unorthodox mechanisms to activate *p21* when Pol II CTD phosphorylating kinases are inhibited, the recruitment of specific elongation factors are defective and mRNA synthesis from certain individual genes is inhibited [60]. From this study testing a handful of genes, a so-called “paradoxical scenario” was suggested, where the transcription of specific stress-response genes through p53 action would escape the global inhibition of mRNA synthesis. Our genome-wide study identifying the group B genes, containing many p53-responsive genes, defines all those genes where the cells seem to use alternative processes to react to UV stress that otherwise would compromise mRNA synthesis in general. Furthermore, the fact that group B genes are not influenced by the depletion of CSB (Figure 8) is in good agreement with previous observations showing that p53-responsive genes do not require functional CSB, while housekeeping genes do [61].

### Pol II is not degraded upon UVB irradiation, but the amount of Pol IIO form increases in parallel with Pol II promoter clearance

Studies investigating the effect of genotoxic stresses on transcription reported two possible pathways by which Pol II protein levels may be affected: either the hyperphosphorylation of the CTD or the degradation of the largest subunit of Pol II [26]. We did not detect any change in the global level of Pol II (Figure 6) therefore it seems that the sublethal UVB irradiation condition used does not induce degradation of the Pol II complex, but rather an active negative regulation of the activity of Pol II. Between 1–4 hours following UVB irradiation, when there is a massive Pol II clearance from the majority of the promoters, the proportion of the Pol IIO form increases, and decreases again to at 6 hours after UVB irradiation, when Pol II presence on the genes is re-established. Thus, our observations seem to be in line with previous studies [62], [63], suggesting that the Pol IIO form is less competent for PIC formation. Nevertheless, at this stage it is not possible to decide whether the increase of the Pol IIO form 1–4 hours following UVB is a cause or a consequence of the above-discussed negative regulatory mechanism.

Surprisingly, none of the antibodies raised against different phosphorylated forms of the CTD hepta-peptide repeat (anti-Ser2, anti-Ser5 or anti-Ser7) revealed increased levels of phosphorylation of the Rpb1 CTD upon UVB stress that would explain the shift from the Pol IIA to the Pol IIO form (Figure 6C). These results suggest a more complex regulation in the CTD modification mechanism(s) that would result in the well detectable and discrete shift from the Pol IIA to the Pol IIO form in western blot assays. Moreover, we detected a quick drop and recovery at 6 hours in Ser7 CTD phosphorylation in parallel with the promoter clearance and recovery of Pol II at the TSSs of the majority of genes. The reduction of the CTD Ser7-P mark between 2 and 4 hours might reflect the above-described negative regulation of Pol II transcription during this time window, and further supports the idea, that Ser7-P is required for gene expression [47].

Taken together all our observations it seems that the massive Pol II clearance on group A genes upon UVB irradiation is not due to Pol II degradation, but due to the incapability of the Pol II complex to initiate transcription. It seems that during TCR the general transcription factor TFIIH is sequestered away from the TSSs by the repair machinery and this in turn would either dismantle genome-wide existing PICs and/or block the formation of new PICs on the majority of the transcribed genes. In contrast, on a small subset of key regulatory genes this negative mechanism does not work either because transcription initiation on these genes is not dependent on the repair-competent form of TFIIH or these genes are repaired much faster than the inhibited genes. Further experiments, will be needed to distinguish amongst all these different exciting possibilities. In conclusion, our study for the first time gives a genome-wide view about the mechanistic details of UVB induced Pol II transcription changes during TCR.

## Materials and Methods

### Cell culture

The MCF7 human cell line (obtained from American Type Culture Collection; reference number HTB-22) was grown in Dulbecco's Modified Eagle Medium (DMEM, Invitrogen) supplemented with 10% foetal calf serum (FCS). The medium contained insulin (0.6 µg/ml) and gentamicin (40 µg/ml). For assessing global and ongoing transcription cells were incubated with 1 mM 5-fluorouridine (Sigma-Aldrich) for 20 minutes. Incorporation of the modified nucleotide was monitored by indirect immunofluorescence using an anti 5-FU antibody from Sigma-Aldrich.

### UVB treatment

For the ChIP and ChIP-seq experiments exponentially growing cells were irradiated with 55 Joules/m^2^ UVB dose at 312 nm wavelength (Vilber T-15M lamp) or with with 55, 100 and 200 Joules/m^2^ (see Figure S1).

### siRNA transfection

siRNAs targeting CSB (ERCC6), GAPD and non-targeting control (L-004888-00-0050, D-001830-10-05, D-001810-10-05, Dharmacon, Thermo Scientific) were transfected to MCF7 cells using Dharmafect Transfection Reagent 1 (T-2001-03, Dharmacon, Thermo Scientific) according to the manufacturers protocol. Cells were harvested for western blot analysis or ChIP 72 hours after the transfection.

### Crystal-violet survival assay

MCF7 cells were plate into a 6 well plate (300 cells/well) one day prior to UV treatment. In the following days, the survived cells will form colonies. 7 days after UV irradiation, the cells were washed with PBS then stained with Crystal violet solution (0.2% crystal violet, 2% ethanol). The staining was removed from the cells with 1% sodium dodecyl sulphate (SDS) containing MQ water. The crystal violet amount in both UV treated and in control samples were measured with spectrophotometer at 595 nm. Results were calculated from biological triplicates

### Slot-blot technique to detect DNA-lesions

Cells were plated in 6 cm plates and UV-treated at 80–90% confluence. After incubation for 1, 2, 4, 6, 8, 16, 24 hours DNA was extracted from samples as well as from non-treated control with SIGMA GenElute Mammalian Genomic DNA Miniprep Kit. DNA content was quantified with Nanodrop. 250 ng of DNA was diluted in 2× SSC (0.3 M NaCl, 0.03M Sodium citrate, pH: 7) buffer from each sample and was transferred to Amersham Hybond-N+ membrane with Slot-blot vacuum chamber. The membrane was treated with Denaturizing buffer (0.5M NaOH, 1.5M NaCl) then Neutralizing buffer (0.5 M Tris-HCl, 1.5 M NaCl). The membrane was then treated according to Western blot protocol: blocked with PBS +2% milk, and then incubated with mouse IgG monoclonal anti-CPD (TDM2) (MBL international corp.) primary antibody overnight in 4°C.

### Western blot assays

MCF7 cells were plated in 6 cm plates prior treatment. At 80–90% confluence, cells were treated with 55 J/m^2^ UVB. The irradiated and control cells 1, 2, 3, 4, 5, 6, hours later after incubation were washed twice with ice-cold PBS containing complete protease inhibitor cocktail (1×), phenylmethylsulfonyl fluoride (PMFS)(0.5 mM), β-glycerophosphate (10 mM), sodium orthovanadate (1 mM), sodium fluoride (20 mM) inhibitors. Cells were scraped in PBS containing protease inhibitor cocktail and frozen-thawed three times. Samples were sonicated for 10 cycles (30 second on, and 30 seconds off) (Bioruptor) then boiled for 5 min in loading solution. Protein samples were separated by a 6–10% SDS-PAGE, transferred and western blot assays were carried out by using standard methods. The following antibodies were used: RNA polymerase II N-terminal H-224× (Santa Cruz), Pol II Ser2, AB: Covance, (MMS-129R), Pol II Ser5 AB: Abcam, (ab5131) Pol II Ser 7 AB: [64], TBP: 3G3 [65], TFIIH subunit (p62): 3C9MAB [66], TFIIB: [67], CDK7: (C-19), sc-529 (Santa Cruz), Tubulin-α: (D-10): sc-135659 (Santa Cruz), GAPD: 6C5 (MAB374, Millipore), CSB: 1CSB- 1A11 [68]. In Figure S1 Phospho-p53 (Ser15) antibody #9284 (Cell Signaling Technology); Phospho-Chk1 (Ser345) (133D3) antibody #2348 (Cell Signaling Technology); Tubulin-α: (D-10) antibody: sc-135659 (Santa Cruz) were used.

### Chromatin immunoprecipitation and qPCR

MCF7 cells were plated in 15 cm dishes. At 80–90% confluence cells were UVB treated as described above. After 1, 2, 3, 4, 5, or 6 hours of incubation cells were quickly washed with PBS, and cross-linked with 1% formaldehyde for 20 minutes at room temperature. ChIP experiments were carried out as described earlier [11]. Validation of the ChIP was performed by quantitative PCR (qPCR) analysis using a Roche LightCycler 480 with Sybr green (Roche) master mix. Oligonucleotides were designed to amplify the promoter regions of the human *Ubc*/NM_021009, *Rplp1*/NM_001003, *p21/cdkn1a*/NM_000389 and *WDR24*/NM_032259 genes. Intergenic region was selected as a negative control region. The ChIP experiments were repeated at least twice, and all the qPCR reactions were done in triplicates.

### High throughput sequencing with Hi-seq 2000

The sample preparation for ChIP-seq was the same as described [11]. To have enough material for sequencing, 5 ChIP samples were pooled together for each time point. Library preparation for sequencing was performed as described by the manufacturer. The generated sequencing data was deposited in the Gene Expression Omnibus (GEO, http://www.ncbi.nlm.nih.gov/gds) database. The 32 base pair tags generated from Hi-seq 2000 were mapped to the human genome (UCSC hg19) using the eland program allowing two mismatches. Only sequences that mapped uniquely to the genome with maximum of two mismatches were used for further analysis. To eliminate non-specific binding sites, we used our control ChIP-seq dataset, which was generated using an antibody that does not recognize any human proteins (GSE34001).

### Genome annotations

Genome annotations were downloaded from the UCSC Genome Browser (https://genome.ucsc.edu/), human genome Build 37 (hg19 assembly). Gene definitions were given by the refseq genes track [69].

### Bioinformatics tools and data-analysis methods

Intergenic regions (around 8000 regions) were selected that are far away from genes about 20 kb and tag numbers were counted on 2 kb interval in the middle of them for all the 7 samples. These values were used as an input for DESeq Bioconductor package, which normalizes the samples based on their median values. (http://www.bioconductor.org/packages/2.9/bioc/vignettes/DESeq/inst/doc/DESeq.pdf). We carried out analyses on all genes found in the refseq database and on 4500 expressed genes, based on the published RNA-seq dataset for MCF-7 cell line [38]. The sequenced ChIP-seq reads represented 36 base pair fragments. To illustrate the entire DNA fragments bound by Pol II, basically before analysis, 3′ end of each ChIP-seq read was extended to 200 bp in the direction of the reads. To generate an average gene profile from ChIP-seq results, Pol II tags were counted on the selected genes with seqMINER software [37] from −1000 bp relative to the TSS of a given gene until the end of EAG +4000 bp regions by dividing these regions into 120 bins (the same number for long as well as for short genes). The ChIP-seq reads were counted in each bin and used to generate the profile. The promoter regions of all refseq genes were divided into 5 bins, while the GB+EAG+4000 regions together were covered by 115 bins. While doing this analysis, the strand orientation is taken in account in order to orientate all analyzed features in the same direction. For heat map generation, Pol II read numbers for the 4500 expressed genes were counted from the seven datasets around the promoter (+/−300 bp around TSS), on the annotated gene body (TSS+100 until the EAG) and downstream of EAG (EAG+4000 bp). During heat map generation, Pol II tag densities were subjected to k-means clustering in order to organize or cluster genes in a same group based upon similar tag enrichment within a defined region. In k-means clustering, number of clusters is fixed and hence the samples are sorted in the clusters based upon the tag enrichment and patterns of Pol II. Cluster and heat map generation was carried out with Cluster 3 and TREEview software.

Cluster:http://bonsai.hgc.jp/~mdehoon/software/cluster/manual/index.html#Top

Treeview: http://sourceforge.net/projects/jtreeview/

### Gene ontology (GO) analyses and gene name conversion

The Database for Annotation, Visualization and Integrated Discovery (D.A.V.I.D.) (http://david.abcc.ncifcrf.gov/home.jsp) was used for GO analyses and gene ID conversion. Further GO enrichment analyses were also performed in Manteia using its statistical module (http://manteia.igbmc.fr/). During the analyses only GO categories with lower than 0.01 p-values (p-value<0.01) were considered as positive results.

## Supporting Information

Figure S1 Effects of sublethal dose of UVB on MCF7 cells. Experiments were carried out to find and characterize the sublethal dose of UVB irradiation for our study. (A) Crystal violet assay was carried out to compare the effect of different doses of UVB on MCF7 cells. The results are represented as a bar chart. The % of surviving cells are shown corresponding to each dose of UVB irradiation (55 J/m^2^, 100 J/m^2^ and 200 J/m^2^) together with the survival of non-treated/control cells. (B) Slot Blot assay was carried out with a CPD specific antibody to test the genotoxicity of 55 J/m^2^ of UVB irradiation. The appearance and the persistence of the CPDs were tested at the indicated time points up to 24 hours after UVB treatment. (C) Western blot assays were carried out to test whether 55 J/m^2^ UVB induces detectable DNA-repair response in MCF7 cells. We tested the induction of two well-characterized regulators of the UV-response at the indicated time points: phospho-Chk1 and phospho-p53, respectively. Tubulin-α was used as loading control.
(PDF)

Figure S2 Global Pol II tag density changes dynamically in the gene body regions of all transcribed genes following UVB irradiation. Mean Pol II read values (Y axis) were calculated for each ChIP-seq sample (as indicated on the x axis) from the GB region of the 4500 expressed genes. Each P value was calculated applying pairwise T-tests between control sample and each of the UVB treated samples (as indicated).
(PDF)

Figure S3 (First and second part): Different Pol II behavior patterns in time after UVB irradiation on selected genes from each subgroup of Figure 4. Pol II tag density is shown on selected representative genes belonging to the different subgroups of Figure 4 (as indicated) as UCSC browser screen shots. The Y axis represents mapped Pol II tag numbers. At the bottom of each panel the structure of the selected gene (with exons, introns, 5′ and 3′ non-translated regions) is represented. Arrows depicted in the genes represent the sense of transcription. On the left of the panels the time after UVB irradiation at which the ChIP was carried out, is indicated. On the top of each panel genomic distances are shown.
(PDF)

Figure S4 CSB depletion using siRNA transfection. Scrambled siRNA (lane 1) and ERCC6 (CSB) siRNA (lane 2) transfection in MCF7 cells was carried out as described in Materials and Methods. Cells were collected 72 hours following transfection and whole cell extracts were made. 20 µg protein extract was loaded on a 10% SDS-PAGE, the gel was blotted and protein levels of ERCC6 (CSB) and actin, as loading control, were detected by western blot analysis using the corresponding antibodies as indicated.
(PDF)

Table S1 Results of gene ontology analyses done with the Manteia software to identify the potential differential gene-function categories for the detected gene groups with distinct Pol II behavior patterns from Figure 3.
(PDF)

Table S2 List of genes represented in Figure 4 of the main text in each clusters (Aa-Ag and B) of the top 4500 expressed genes. This file also contains the list of the selected repair genes in each clusters (Repair a–e) represented in Figure 5 of the main text. Refseq gene entries were used as gene names.
(XLSX)
