# Supplementary figures and images for: UVB Induces a Genome-Wide Acting Negative Regulatory Mechanism That Operates at the Level of Transcription Initiation in Human Cells
Source: PLoS Genet. 2014 Jul 24;10(7):e1004483. doi: 10.1371/journal.pgen.1004483 (PMC4109906; doi:10.1371/journal.pgen.1004483)

**A**

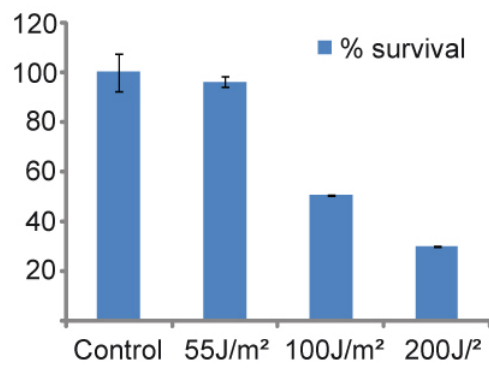

**B**

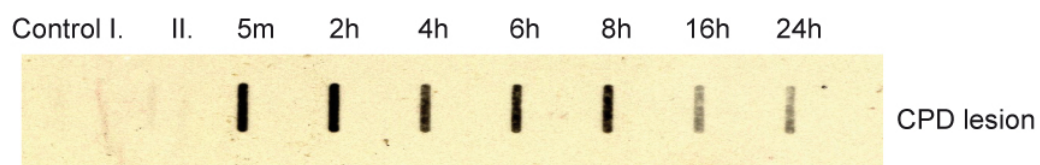

**C**

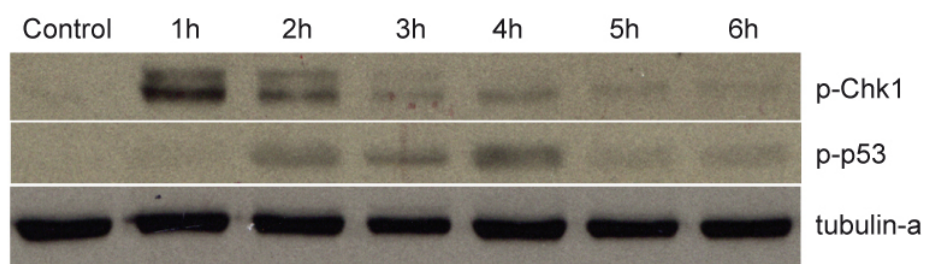

**Figure S1**

Supplement: Figure S1 — Effects of sublethal dose of UVB on MCF7 cells. Experiments were carried out to find and characterize the sublethal dose of UVB irradiation for our study. (A) Crystal violet assay was carried out to compare the effect of different doses of UVB on MCF7 cells. The results are represented as a bar chart. The % of surviving cells are shown corresponding to each dose of UVB irradiation (55 J/m2, 100 J/m2 and 200 J/m2) together with the survival of non-treated/control cells. (B) Slot Blot assay was carried out with a CPD specific antibody to test the genotoxicity of 55 J/m2 of UVB irradiation. The appearance and the persistence of the CPDs were tested at the indicated time points up to 24 hours after UVB treatment. (C) Western blot assays were carried out to test whether 55 J/m2 UVB induces detectable DNA-repair response in MCF7 cells. We tested the induction of two well-characterized regulators of the UV-response at the indicated time points: phospho-Chk1 and phospho-p53, respectively. Tubulin-α was used as loading control. (PDF) [file pgen.1004483.s001.pdf]

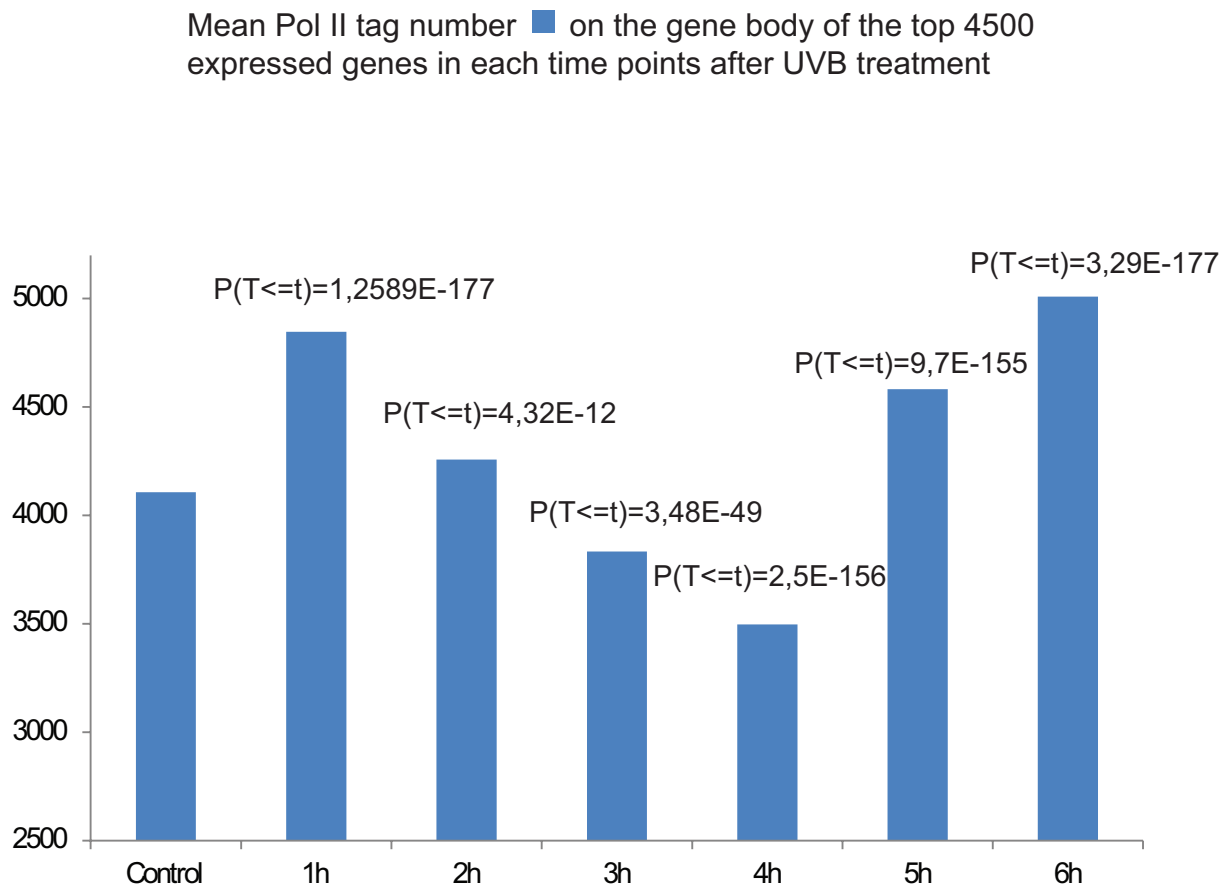

**Figure S2**

Supplement: Figure S2 — Global Pol II tag density changes dynamically in the gene body regions of all transcribed genes following UVB irradiation. Mean Pol II read values (Y axis) were calculated for each ChIP-seq sample (as indicated on the x axis) from the GB region of the 4500 expressed genes. Each P value was calculated applying pairwise T-tests between control sample and each of the UVB treated samples (as indicated). (PDF) [file pgen.1004483.s002.pdf]

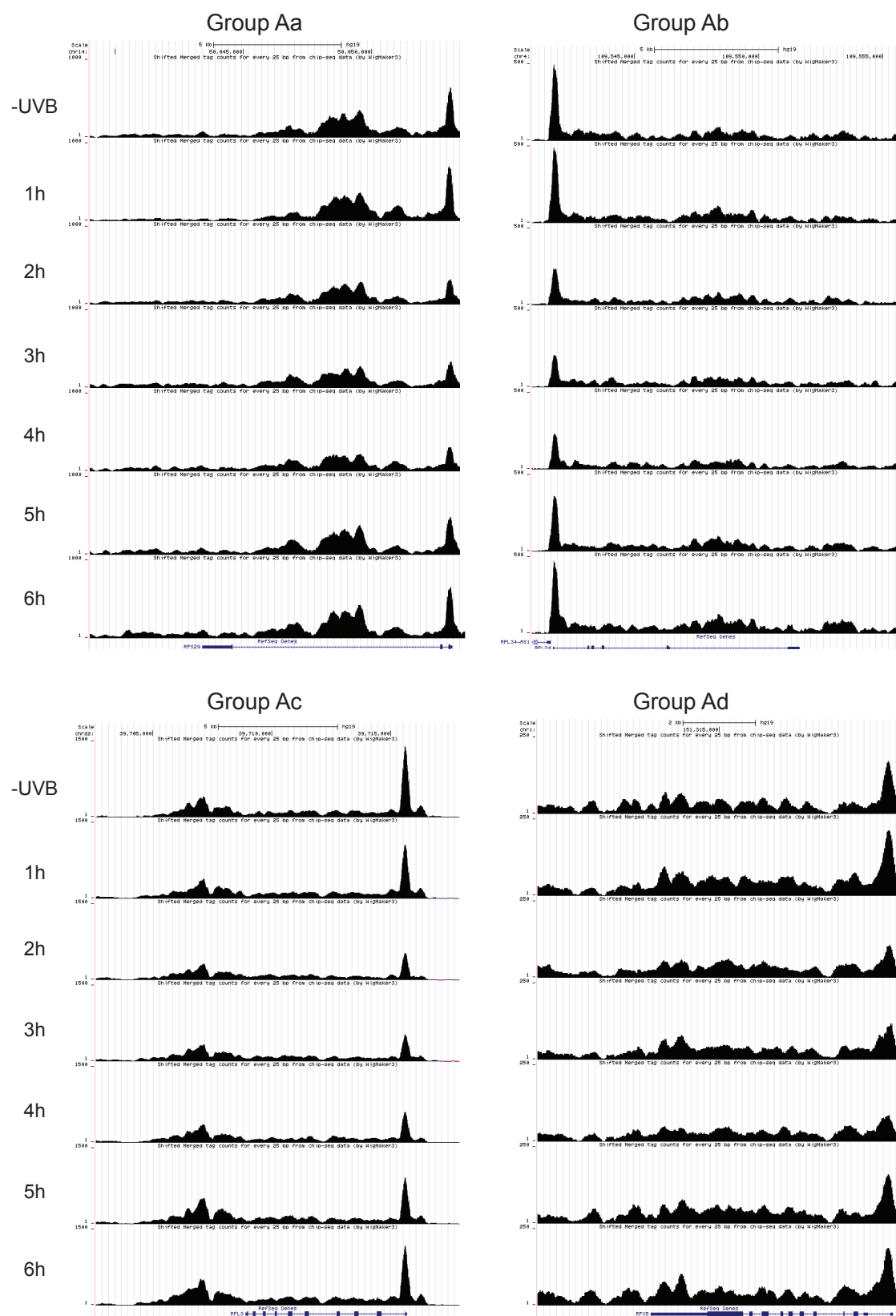

Figure S3 (first part)

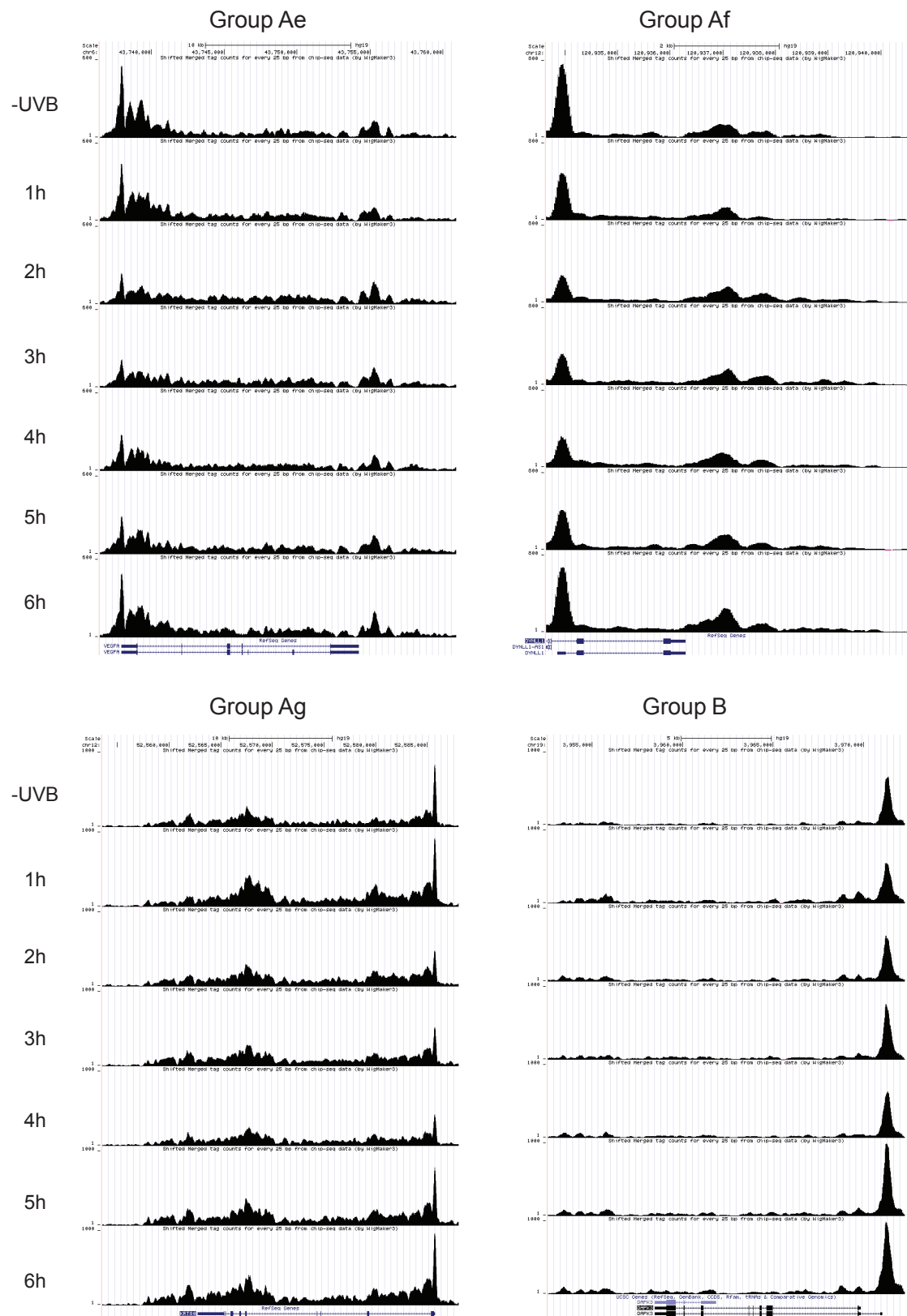

Figure S3 (Second part):

Supplement: Figure S3 — (First and second part): Different Pol II behavior patterns in time after UVB irradiation on selected genes from each subgroup of Figure 4. Pol II tag density is shown on selected representative genes belonging to the different subgroups of Figure 4 (as indicated) as UCSC browser screen shots. The Y axis represents mapped Pol II tag numbers. At the bottom of each panel the structure of the selected gene (with exons, introns, 5′ and 3′ non-translated regions) is represented. Arrows depicted in the genes represent the sense of transcription. On the left of the panels the time after UVB irradiation at which the ChIP was carried out, is indicated. On the top of each panel genomic distances are shown. (PDF) [file pgen.1004483.s003.pdf]

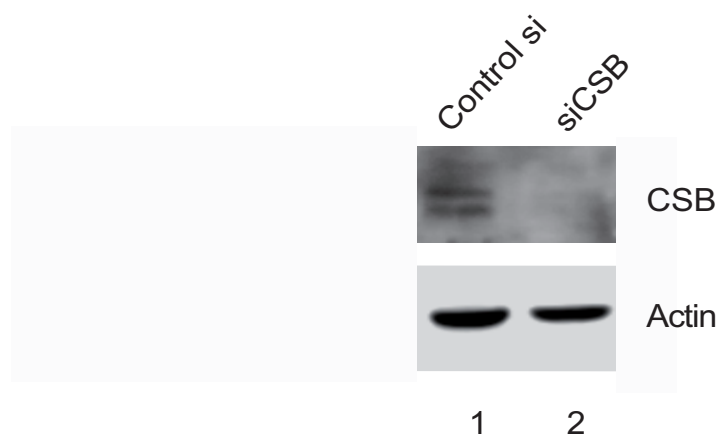

**Figure S4:**

Supplement: Figure S4 — CSB depletion using siRNA transfection. Scrambled siRNA (lane 1) and ERCC6 (CSB) siRNA (lane 2) transfection in MCF7 cells was carried out as described in Materials and Methods. Cells were collected 72 hours following transfection and whole cell extracts were made. 20 µg protein extract was loaded on a 10% SDS-PAGE, the gel was blotted and protein levels of ERCC6 (CSB) and actin, as loading control, were detected by western blot analysis using the corresponding antibodies as indicated. (PDF) [file pgen.1004483.s004.pdf]
