# Supplementary material for: UVB Induces a Genome-Wide Acting Negative Regulatory Mechanism That Operates at the Level of Transcription Initiation in Human Cells
Source: PLoS Genet. 2014 Jul 24;10(7):e1004483. doi: 10.1371/journal.pgen.1004483 (PMC4109906; doi:10.1371/journal.pgen.1004483)
Supplement: Table S1 — Results of gene ontology analyses done with the Manteia software to identify the potential differential gene-function categories for the detected gene groups with distinct Pol II behavior patterns from Figure 3. (PDF) [file pgen.1004483.s005.pdf]

MANTEIA

Group	GO term	p-value
Aa	cellular protein metabolic process	1,53E-09
	mRNA metabolic process	6,81E-08
	cellular component organization at cellular level	6,88E-08
Ab	negative regulation of macromolecule metabolic process	8,90E-14
	negative regulation of transcription, DNA-dependent	5,34E-10
	regulation of transcription from RNA polymerase II promoter	3,22E-08
Ac	cellular macromolecular complex subunit organization	1,07E-28
	translational elongation	8,58E-28
	translational termination	5,90E-26
Ad	mRNA metabolic process	2,17E-17
	cellular macromolecule metabolic process	1,08E-11
	primary metabolic process	4,05E-10
Ae	cellular metabolic process	3,15E-18
	translation	1,64E-14
	primary metabolic process	9,42E-14
Af	translation	9,46E-21
	signal transduction	4,87E-11
	mRNA metabolic process	3,01E-18
Ag	energy derivation by oxidation of organic compounds	9,97E-08
	response to UV	1,36E-05
	negative regulation of macromolecule metabolic process	7,16E-06
B	regulation of cellular metabolic process	5,25E-07
	apoptotic process	3,93E-05
	RNA metabolic process	1,92E-05

Table S1
